# *DJExpress*: An Integrated Application for Differential Splicing Analysis and Visualization

**DOI:** 10.3389/fbinf.2022.786898

**Published:** 2022-02-24

**Authors:** Lina Marcela Gallego-Paez, Jan Mauer

**Affiliations:** BioMed X Institute (GmbH), Heidelberg, Germany

**Keywords:** alternative splicing, splicing aberrations, differential splicing analysis, cancer splicing, The Cancer Genome Atlas Program (TCGA), GTEx database

## Abstract

RNA-seq analysis of alternative pre-mRNA splicing has facilitated an unprecedented understanding of transcriptome complexity in health and disease. However, despite the availability of countless bioinformatic pipelines for transcriptome-wide splicing analysis, the use of these tools is often limited to expert bioinformaticians. The need for high computational power, combined with computational outputs that are complicated to visualize and interpret present obstacles to the broader research community. Here we introduce *DJExpress*, an R package for differential expression analysis of transcriptomic features and expression-trait associations. To determine gene-level differential junction usage as well as associations between junction expression and molecular/clinical features, *DJExpress* uses raw splice junction counts as input data. Importantly, *DJExpress* runs on an average laptop computer and provides a set of interactive and intuitive visualization formats. In contrast to most existing pipelines, *DJExpress* can handle both annotated and *de novo* identified splice junctions, thereby allowing the quantification of novel splice events. Moreover, *DJExpress* offers a web-compatible graphical interface allowing the analysis of user-provided data as well as the visualization of splice events within our custom database of differential junction expression in cancer (DJEC DB). DJEC DB includes not only healthy and tumor tissue junction expression data from TCGA and GTEx repositories but also cancer cell line data from the DepMap project. The integration of DepMap functional genomics data sets allows association of junction expression with molecular features such as gene dependencies and drug response profiles. This facilitates identification of cancer cell models for specific splicing alterations that can then be used for functional characterization in the lab. Thus, *DJExpress* represents a powerful and user-friendly tool for exploration of alternative splicing alterations in RNA-seq data, including multi-level data integration of alternative splicing signatures in healthy tissue, tumors and cancer cell lines.

## Introduction

Splicing of pre-mRNA is a crucial process in eukaryotic gene expression regulation. In addition to canonical splicing, which leads to the inclusion of constitutive exons into the mature mRNA, the transcriptome is subjected to alternative splicing. Alternative splicing can give rise to multiple protein-coding isoforms from a single pre-mRNA and thus represents a major determinant for proteome diversity. Approximately 92%–94% of human genes generate alternatively spliced transcripts, often with tissue-specific regulation (Wang et al., 2008; Barbosa-Morais et al., 2012). Alternative splicing is involved in a variety of cellular processes, such as cell proliferation, differentiation, migration and survival (Paronetto et al., 2016; Gallego-Paez et al., 2017). Emerging data indicate that alternative splicing plays a critical role in the pathogenesis of many diseases, including several molecular subtypes of cancer (Oltean and Bates, 2014; Scotti and Swanson, 2016; Jiang and Chen, 2021). Interrogating such splicing abnormalities can facilitate identification of disease drivers, drug resistance mechanisms, and molecules capable of regulating pathological splicing events. Thus, exploration of alternative and aberrant splicing phenotypes promises to shed light on novel aspects of health and disease.

The recent release of transcriptome-wide RNA sequencing (RNA-seq) data repositories such as The Cancer Genome Atlas (TCGA) (Tomczak et al., 2015) and the Genotype-Tissue Expression (GTEx) project (Lonsdale et al., 2013) have lifted alternative splicing analysis opportunities to an unprecedented level. However, a unified and accessible analysis strategy for this data has largely been missing.

The gradual development of RNA-seq technologies and cost-effective alternative splicing studies at the transcriptome level has allowed the parallel evolution of bioinformatic tools for splicing quantification and visualization. Most of these tools rely on two main computational approaches: 1) quantification of the Percent Spliced-In (PSI) metric, which uses the ratio between exon-exon junction spanning sequencing reads that provide evidence for the inclusion or exclusion of an alternatively spliced region [e.g., rMATS (Shen et al., 2014), MISO (Katz et al., 2010), SUPPA (Alamancos et al., 2015), SplAdder (Kahles et al., 2016), psichomics (Saravia-Agostinho and Barbosa-Morais, 2019), AltAnalyze (Emig et al., 2010), SpliceSeq (Ryan et al., 2012), VAST-TOOLS (Irimia et al., 2014), MAJIQ (Vaquero-Garcia et al., 2016), LeafCutter (Li et al., 2018) and Whippet (Sterne-Weiler et al., 2018)], and 2) quantification and de-convolution of the entire set of reads aligned to the gene to estimate transcript isoform abundance (e.g., Cufflinks (Trapnell et al., 2010), RSEM (Li and Dewey, 2011), Sailfish (Patro et al., 2014), Salmon (Patro et al., 2017) and Kallisto (Bray et al., 2016)) (see Table 1 for a comparison of these tools). Although these bioinformatic tools have propelled transcriptome-wide alternative splicing analysis forward, they suffer from significant limitations. These include the need for high computational resources and bash-based operation, restrictions of input file formats, incomplete transcriptome annotation and consequently inaccurate transcript/PSI quantification. Furthermore, these tools suffer from complex static graphical outputs that are complicated to visualize and interpret or lack the option for association of splicing phenotypes to clinical or molecular data. These caveats are obstacles for a straight-forward interpretation of the biological and physiological relevance of alternative splicing in disease. Thus, despite the large variety of available tools, there is still a high demand for easy-to-use alternative splicing analysis strategies that can incorporate comprehensive data visualization and integration with external sample traits.

**TABLE 1:** Feature comparison between *DJExpress* and other existing splicing analysis tools.

Tool	GUI	User-selected alignment method	Non-annotated junctions supported	Splicing pattern visualization	Downstream trait association
DJExpress	Yes	Yes	Yes	Yes	Yes
MAJIQ	Yes	Yes	Yes	Yes	No
Psichomics	Yes	Yes	No	Yes	Yes
AltAnalize	Yes	Yes	No	Yes	Yes
LeafCutter	Yes	No	Yes	Yes	Yes
SplAdder	No	Yes	Yes	Yes	No
rMATS	No	Yes	Yes	No	No
SpliceSeq	Yes	No	No	Yes	No
Whippet	No	No	Yes	Yes	No
JunctionSeq	No	No	Yes	Yes	No
MISO	No	No	No	Yes	No
SUPPA	No	Yes	No	No	No
Cufflinks	No	No	Yes	No	No
Salmon	No	Yes	No	No	No
RSEM	No	Yes	No	No	No
Sailfish	No	No	No	No	No
VAST-TOOLS	No	No	No	No	No
Kallisto	No	No	No	No	No

Here we introduce a novel differential junction expression analysis pipeline, *DJExpress*, which is an R package for analysis of transcriptomic features and expression-trait associations. *DJExpress* runs on an average laptop computer (Supplementary Figure S1) and provides a set of interactive and intuitive visualization formats. *DJExpress* uses raw splice junction counts—derived from STAR aligner (Dobin et al., 2013) or other junction quantification algorithms—as input data to determine gene-level differential junction usage. The statistical approaches implemented by *DJExpress* include empirical Bayesian procedures to assess differential junction expression between experimental conditions and junction-level t-statistics tests to determine differences between each junction and all other junctions within the same gene.

In contrast to the majority of existing pipelines, *DJExpress* can handle both annotated and *de novo* identified splice junctions, thereby allowing the characterization of novel splice events. Moreover, through gene-level differential junction usage calculation, *DJExpress* identifies associations between junction expression and molecular/clinical features using large matrix operations. An additional more advanced feature of *DJExpress* involves weighted junction co-expression network analysis (JCNA). JCNA-derived junction expression modules can be correlated with phenotypes of interest, thereby allowing differential splicing analysis on a systemic scale. For downstream processing, JCNA outputs can be exported in a format compatible with network visualization tools such as VisANT and Cytoscape (Shannon et al., 2003; Hu et al., 2004).

In addition to these locally accessible features, *DJExpress* offers a web-compatible graphical interface for the analysis of user-provided data as well as the visualization of DJEC DB, a custom database of cancer-specific splicing profiles and their association to external traits from tumor samples and cancer cell lines. DJEC DB includes not only TCGA and GTEx data, but also cancer cell line data from the Cancer Dependency Map (DepMap^1^) project. The integration of DepMap data allows association of junction expression with functional genomics features such as gene dependencies and drug response profiles. This facilitates identification of cancer cell models for specific splicing alterations that can then be used for functional characterization in the lab.

Taken together, *DJExpress* represents a novel and versatile tool to analyze and explore alternative splicing phenotypes in health and disease.

## Methods

### Differential Junction Expression Module

**FIGURE 1:**
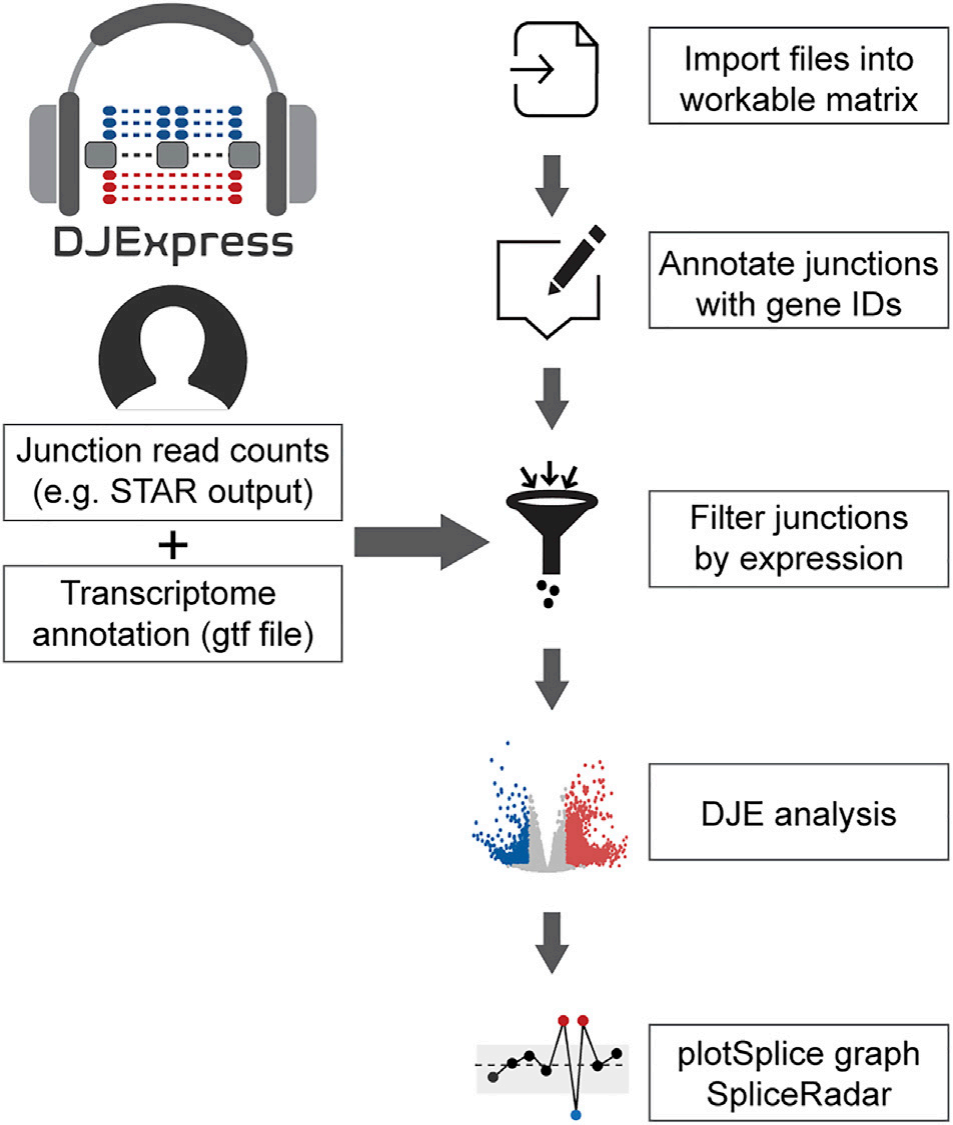
General workflow of the DJE analysis module in *DJExpress*. Junction quantification files (e.g., SJ.out.tab files from STAR aligner) and transcriptome annotation files (gft file format) are provided by the user as input. Junctions are then annotated with their corresponding genes and filtered based on user-defined expression cutoffs. Differential junction expression is then calculated between experimental conditions. Significant differences in junction usage can be interactively visualized using the gene-wise PlotSplice graph. When external trait data is provided, the DJE module can identify significant junction-trait associations that can be further visualized using SpliceRadar plots.

**FIGURE 2:**
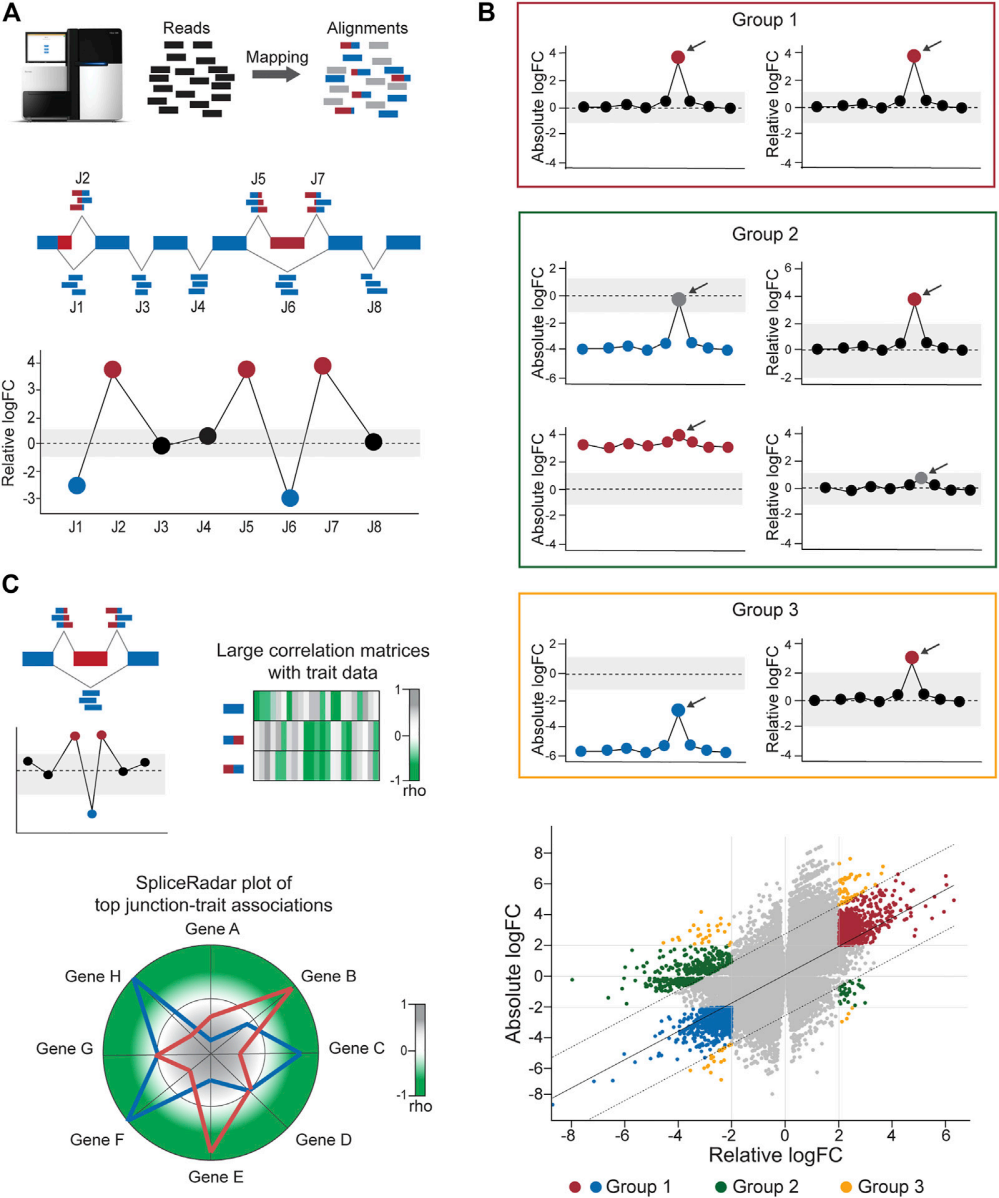
Calculation of differential junction expression using the DJE module. **(A)** After alignment and quantification of RNA-seq reads supporting exon–exon junctions, differential junction expression is analyzed and depicted using the gene-wise splice plot visualization method. The schematic shows 8 junctions (J1-J8) in hypothetical gene, where each junction is plotted along the *x*-axis and ordered by genomic coordinate position. Relative log-fold change values (logFC), which indicate the difference between the expression of the target junction vs the average junction expression in the gene is shown in the *y*-axis. Junctions with logFC values above a user-defined threshold (absolute logFC of 1.0 in the example) are considered as differentially used and colored blue or red in case of downregulation and upregulation, respectively. **(B)** *DJExpress* determines alternatively spliced transcript regions based on both, alterations in their expression levels compared to the average expression of other junctions the same gene (differential usage, based on relative logFC) and alterations in junction abundance between experimental conditions (differential expression, based on absolute logFC). Junctions are then classified into four main groups. Group 0 corresponds to junctions without differential expression or differential usage and is visually represented as grey points in the scatter plot. Group 1 (red box and red/blue points in the scatter plot) comprises junctions with similar values of absolute and relative logFCs which reflects changes in splicing patterns between experimental conditions without confounding alterations in the total expression of the gene. Group 2 (green box and green points in the scatter plot) represents junctions with differential expression but no differential usage or vice-versa, which indicates the presence of altered total gene expression levels between conditions that explain observed differences. Group 3 (orange box and orange points in the scatter plot) designates junctions with significant but dissimilar levels of relative and absolute logFCs, indicating the presence of both, total gene expression and local splicing changes. Relative vs absolute logFC plots are produced within the output of the DJE module, where junctions are classified into specific groups according to the significance of their logFC values and their position inside or outside of the distribution by ≥2 standard deviations. Arrows indicate example target junctions. **(C)** When external sample trait data (e.g., clinical or molecular data) are provided by the user, *DJExpress* can identify significant junction-trait associations within a target experimental condition using either correlation analysis, ANOVA test or linear regression models. If correlation is selected by the user (as in the depicted example), the results are used to construct heatmap or SpliceRadar plots with target splice junctions (e.g., inclusion junctions (red) and exclusion junction (blue) in an exon skipping event). In the case of SpliceRadars, positive correlation coefficients are located within the outer region (green) and negative correlation coefficients are found within the inner region (grey) of the radar chart, allowing the visual inspection of multivariate trait associations to user-selected alternative splicing events.

The data analysis workflow in the DJE module is depicted in Figure 1. For differential junction expression (DJE) and junction co-expression network analysis (JCNA), *DJExpress* uses quantified raw reads aligned to exon-exon junction loci and the transcriptome annotation as the primary input. Mapped and quantified junction reads are typically generated from FASTQ or BAM files using common RNA-seq alignment/quantification tools [e.g., STAR (Dobin et al., 2013), TopHat (Trapnell et al., 2009), MapSplice (Wang et al., 2010), Rsubread (Liao et al., 2019)] (Figure 2A). Following the statistical principles in limma Bioconductor package (Law et al., 2014; Ritchie et al., 2015), *DJExpress* first tests for differential expression of genomic features (here splice junction regions) using an initial input matrix of read count values as rows and sample ids as columns. Count data is then transformed to log2-counts per million (logCPM), and observation-level weights based on mean-variance relationship are computed (using the *voom* function from *limma*). Users can decide at this point whether to keep the default expression threshold for filtering junctions prior to hypothesis testing (10 minimum of read count mean per junction) or to adjust the threshold based on the mean-variance trend. A linear model is then fit per junction using a provided experimental design, and empirical Bayes moderated *t*-statistics are implemented to assess the significance level of the observed expression changes.

The linear model framework of *limma* is also used in parallel to calculate differential junction usage, where significant differences in log-fold changes in the fit model between junctions from the same gene are tested (using the *diffSplice* function from *limma*). *DJExpress* thereby identifies alternatively spliced regions in transcripts based on two main features of splice junction expression: 1) Quantitative changes in the abundance of individual junctions between experimental groups, and 2) Differences in their expression levels compared to the average expression of other junctions in the gene.

Following these criteria, splice junctions are classified based on their absolute log-fold change (e.g., experimental condition A vs B) and their relative log-fold change (target junction vs all other junctions in the gene) in one of the following expression groups (Figure 2B):

Group 0: Junctions without differential expression or differential usage.
Group 1: Junctions with equal levels of differential expression and differential usage, reflecting changes in splicing patterns between experimental conditions (in this case, both absolute and relative log-fold change values are similar, if not the same).
Group 2: Junctions with differential expression but no differential usage or vice versa, implying the occurrence of generalized changes in expression across the gene, rather than the presence of a differentially spliced region (in this case, either the absolute or relative log-fold change value is not significant).
Group 3: Junctions with divergent levels of differential expression and differential usage, indicating concomitant changes in splicing and total gene expression (in this case, the absolute and relative log-fold change values can substantially vary from each other).

One of the main features of DJE module’s approach is the incorporation of an interactive gene-wise junction representation (Figure 2A). This approach facilitates straight-forward visual inspection of differential splicing across the gene and exploration of supplementary information about each junction’s expression. This includes the above-mentioned classification based on absolute and relative log-fold change patterns, basic statistics on expression levels (e.g., mean and median expression in each experimental condition, number of samples expressing the junction, etc.) as well as the identification of non-annotated and condition-specific junctions. The latter are also called “neojunctions” in the *DJExpress* pipeline, referring to junctions detected in the tested condition but are not found in the control condition.

### Junction-Trait Association Module

Further exploration of the potential physiological relevance of alternative splicing is possible through the association of junction expression to external sample traits (e.g., clinical or molecular data). Significant junction-trait linkages are determined by large matrix operations including correlation analysis, ANOVA test or linear regression models [using *cor and bicor* from *WGCNA* (Langfelder and Horvath, 2008) and *Matrix_eQTL_engine* from *MatrixEQTL* (Shabalin, 2012)]. The top significant association can be visualized though heatmap plots or alternatively, using the SpliceRadar plot format (Figure 2C), where the coefficient of top-ranked correlations is used to map each junction-trait association within a radar chart. This graphical concept allows the users to simultaneously visualize relevant associations between the expression of selected junctions (e.g., the top most differentially expressed junctions or a subset of junctions within a target gene) and external traits, as well as to elucidate expression-trait patterns shared among junctions of interest with potential biological relevance.

### Junction Co-Expression Network Analysis Module

A widely used approach for describing correlation networks in systems biology is the weighted gene co-expression network analysis (WGCNA, Langfelder and Horvath, 2008). WGCNA is a screening method based on pairwise correlations between features in gene expression data. This approach allows the identification of clusters (or modules) of highly correlated genes, intramodular hub genes and representative module eigengenes (MEs). These can be used in the estimation of module membership values for each gene as well as in association analyses between modules and to external sample traits. This technique has been frequently implemented for the assessment of gene-network signatures and for the identification of functional pathways and candidate molecular biomarkers, integrating gene expression and clinical/molecular data from physiological and disease conditions (Oldham et al., 2008; Presson et al., 2008; Ma et al., 2017; Vieira et al., 2019).

**FIGURE 3:**
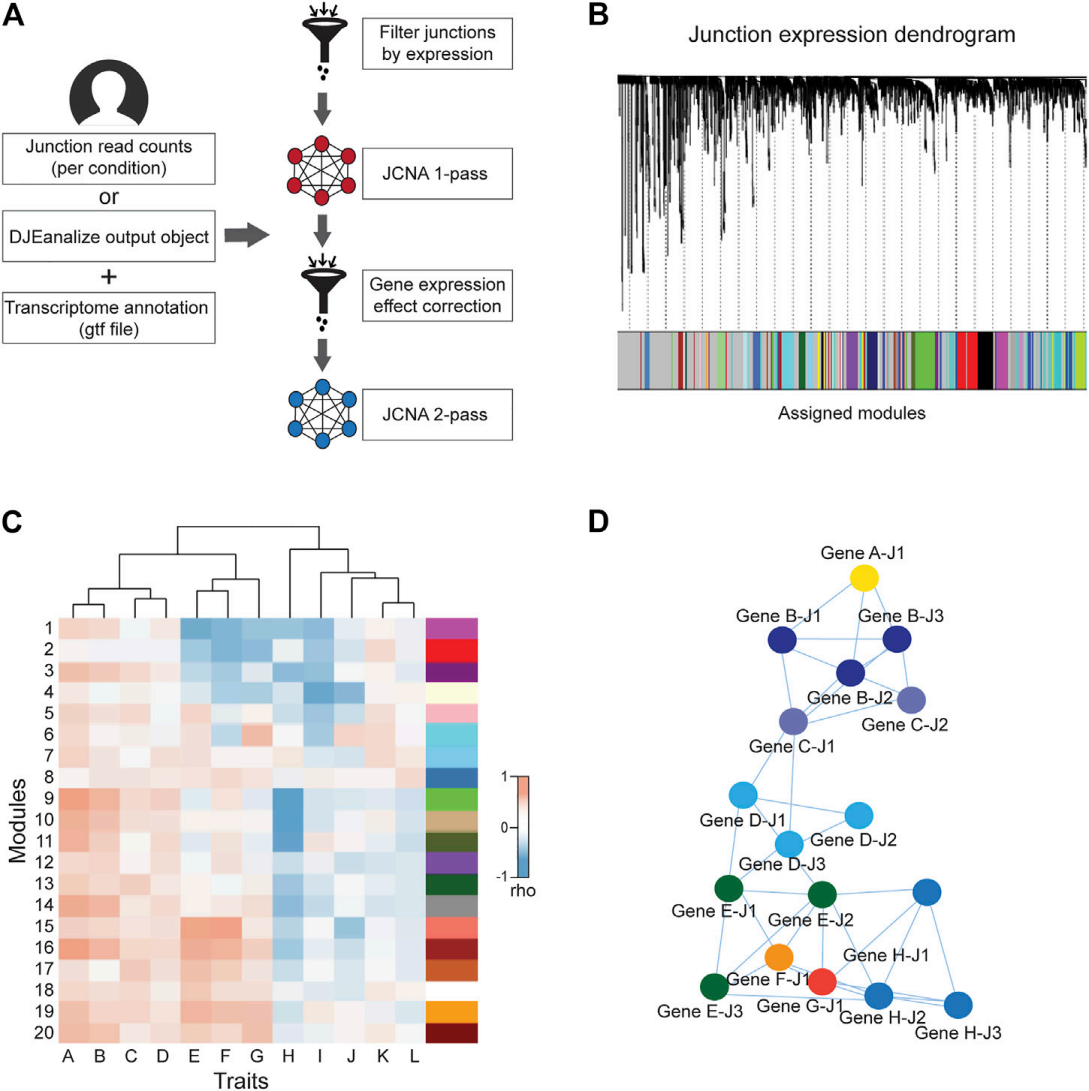
General workflow of JCNA module in *DJExpress*. **(A)** For the *DJExpress* JCNA module, the user needs to provide junction read counts (or the output of the *DJEanalize* function) and a transcriptome annotation file. After removing outlier samples and lowly expressed junctions, a first round of co-expression analysis is performed where junction modules and module/junction vs trait associations are calculated. The user can continue into a second round of network construction, where co-expression analysis and trait association is produced using gene expression data. This information is used to identify and remove junction-trait correlations from the network that reflect gene expression-based associations. The remaining junction set is used to re-construct junction co-expression modules and module-trait correlations. **(B)** Dendrogram schematic of clustered junctions with assigned modules based on a dissimilarity measure (1-TOM) as described for WGCNA (Langfelder and Horvath, 2008). **(C)** Heatmap schematic of correlations between junction module eigengenes (MEs) and different sample traits. **(D)** Schematic representation of interaction networks of junctions within a co-expression module that can be produced using Cytoscape or VisANT visualization tools. Junctions belonging to the same gene are indicated by the same color.

The weighted junction co-expression network analysis module (JCNA) in *DJExpress* provides an implementation of *WGCNA* algorithms (version 1.70.3, Langfelder and Horvath, 2008) in the context of splice junction expression when sufficient sample size is provided (≥15 samples within single experimental conditions as suggested in the *WGCNA* guidelines) (Figure 3A). JCNA initiates with a data pre-processing step where outlier samples (clustered using the average linkage method) and lowly expressed junctions are removed to ensure high confidence network construction. Correlation matrices (e.g., using Pearson, Spearman or the default biweight midcorrelation) (Wilcox, 2012) are then built for all pair-wise junctions. The full network is subsequently specified by a weighted adjacency matrix calculated with an appropriate soft threshold power (Zhang and Horvath, 2005). Summary plots of a network topology analysis are produced by JCNA (following *WGCNA* guidelines) to aid users in the selection of the soft-thresholding power around which scale-free topology in the junction network is achieved.

Additional parameters such as minimum module size, module detection sensitivity or cut height of the hierarchical clustering dendrogram for module definition can be introduced for junction module identification (Figure 3B). Calculation of MEs is also possible, where expression patterns of all junctions in a module are summarized into a single expression profile. This measure is then used in the correlation analysis with sample traits. Notably, ME calculation reduces the computational burden of multiple testing, which otherwise can be exceedingly high since junction quantification datasets usually comprise millions of expression features.

Users can either keep the output of a 1-pass JCNA or can continue into a second round of network construction. During this 2-pass JCNA, the gene expression-specific effect within junction modules is subtracted. This is particularly relevant in the context of junction-trait associations, since a considerable number of co-expressing junctions are expected to cluster into single modules as a result of intrinsic associations at the gene expression level. Here, 2-pass JCNA improves the identification of true co-splicing signatures, since junctions from the same gene or from highly correlated genes tend to cluster without any specific association to splicing.

For 2-pass JCNA, gene expression-based networks including correlations with a user-selected sample trait are calculated (Figure 3C). The absolute value of junction significance, which represents the correlation coefficient between a given junction and the selected trait is plotted as a function of the corresponding gene significance. Junctions outside of the distribution by ≥ 2 standard deviations (showing no correlation between junction and gene significance for trait) are kept for network re-construction. Thus, 2-pass JCNA strategy allows the user to further explore associations between molecular/clinical traits and modules of co-expressed splicing events that can be defined once gene expression-related junction co-expression is identified and removed from the network.

Furthermore, as in the case of *WGCNA* pipeline, the resulting junction modules from JCNA can be also exported to network graphical tools such as Cytoscape or VisANT for further visual exploration and customization (Figure 3D).

### Run Time and Memory Benchmarks

For run time and memory consumption benchmarks of function within the DJE module (*DJEimport*, *DJEannotate*, *DJEprepare* and *DJEanalyze*), we used STAR-derived junction quantification files from the TCGA COADREAD tumor sample cohort. *DJExpress* pipeline was applied 10 times on two cores of a macOS X 11.6.1 system with 2.3 GHz Quad-Core Intel Core i5 processor and 16 GB of memory, RStudio Desktop 1.4. 1106 and R 4.0.5. Each run was performed on datasets with increasing number of samples (e.g., 10, 20, 40, 60, 80, 100, 200, 400,600, 800, 1000) and 100,000 randomly retrieved splice junctions. For the differential junction expression analysis using *DJEanalyze*, samples were randomly divided into two groups using Bernoulli distributed values with a 50% probability of success (Supplementary Figure S1).

### Data Collection for Differential Junction Expression in Cancer Database

**FIGURE 4:**
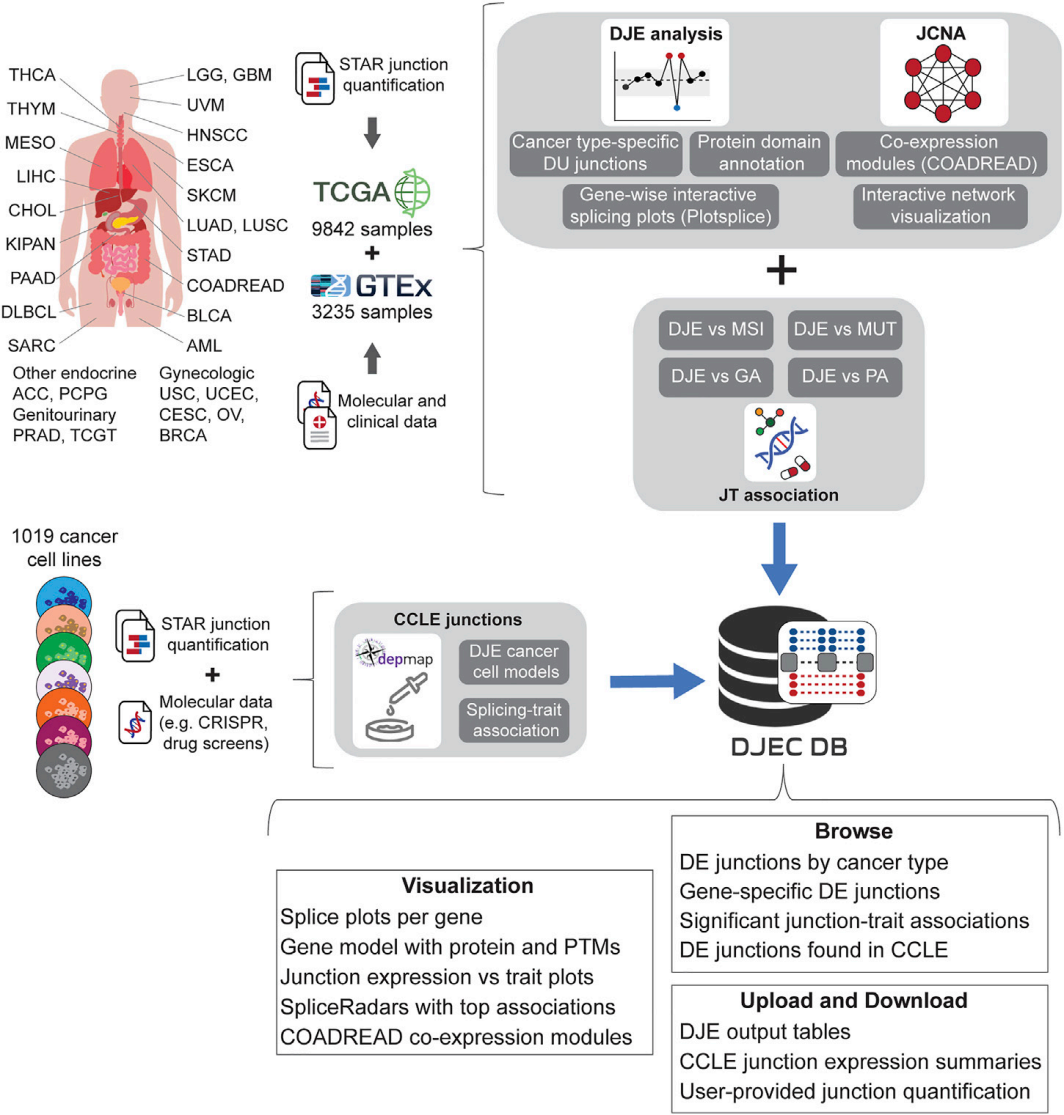
Schematic representation of DJEC DB data generation. DJEC DB takes STAR-based junction quantification across cancer tissue types and normal tissue extracted from the Cancer Genome Atlas (TCGA) and the Genotype-Tissue Expression (GTEx) database respectively. Significant differences in junction usage between tumor and normal tissues were produced following DJE module pipeline. Cancer type-specific DJE with supplementary information (e.g., statistics summary, absolute vs relative logFC group, etc.) as well as gene-wise splice graphs and domain-annotated gene models with the position of user-selected junctions can be also visualized. Differentially expressed junctions in COADREAD were used as example data for junction co-expression network analysis (JCNA). Associations between DJE and TCGA-associated trait data including microsatellite instability (MSI), mutations (MUT), genomic alterations (GA) and pathway alterations (PA) can be explored within the “JT association” section. Junction quantification data from cell lines within DepMap repository was also introduced in the “CCLE junctions” section, allowing the user to identify cancer cell models for specific splicing alterations and splicing-trait associations that can be used for functional characterization of splicing-trait associations in the lab (TCGA tumor type abbreviation codes are as follows: ACC, adrenocortical carcinoma; BLCA, bladder urothelial carcinoma; BRCA, breast invasive carcinoma; CESC, cervical squamous cell carcinoma and endocervical adenocarcinoma; CHOL, cholangiocarcinoma; COAD, colon adenocarcinoma; DLBC, diffuse large B-cell lymphoma; ESCA, esophageal carcinoma; GBM, glioblastoma multiforme; HNSC, head and neck squamous cell carcinoma; KICH, chromophobe renal cell carcinoma; KIRC, clear cell renal clear cell carcinoma; KIRP, papillary renal cell carcinoma; LAML, acute myeloid leukemia; LGG, lower-grade glioma; LIHC, hepatocellular carcinoma; LUAD, lung adenocarcinoma; LUSC, lung squamous cell carcinoma; MESO, mesothelioma; OV, ovarian serous adenocarcinoma; PAAD, pancreatic adenocarcinoma; PCPG, phaeochromocytoma and paraganglioma; PRAD, prostate adenocarcinoma; READ, rectal adenocarcinoma; SARC, adult soft tissue sarcoma; SKCM, cutaneous melanoma; STAD, stomach adenocarcinoma; TGCT, testicular germ cell tumor; THCA, thyroid carcinoma; THYM, thymoma; UCEC, uterine corpus endometrial carcinoma; UCS, uterine carcinosarcoma; UVM, uveal melanoma).

Using the pipelines described for the DJE and JCNA modules, we generated DJEC DB, a custom database of cancer-specific splicing profiles and their association to external traits from tumor samples and cancer cell lines (Figure 4). DJEC DB can be accessed through a graphical interface based on the *shiny* package (version 1.6.0) and includes healthy and tumor tissue data for 9,842 human samples across 32 different tumor types from TCGA, 3,235 normal post-mortem tissue samples from GTEx and 1,019 cancer cell lines from the DepMap Project.

Alignment of GTEx and TCGA RNA-seq data sets to the GRCh37 reference genome and subsequent splice junction quantification, as well as removal of low-quality tissue samples was previously done (Kahles et al., 2018) using the STAR aligner tool with the following arguments:

STAR --genomeDir GENOME --readFilesIn READ1 READ2 --runThreadN 4 --outFilterMultimapScoreRange 1 --outFilterMultimapNmax 20 --outFilterMismatchNmax 10 --alignIntronMax 500000 --alignMatesGapMax 1000000 --sjdbScore 2 --alignSJDBoverhangMin 1 --genomeLoad NoSharedMemory --limitBAMsortRAM 70000000000 --readFilesCommand cat --outFilterMatchNminOverLread 0.33 --outFilterScoreMinOverLread 0.33 --sjdbOverhang 100 --outSAMstrandField intronMotif --outSAMattributes NH HI NM MD AS XS --sjdbGTFfile GENCODE_ANNOTATION --limitSjdbInsertNsj 2000000 --outSAMunmapped None --outSAMtype BAM SortedByCoordinate --outSAMheaderHD @HD VN:1.4 --outSAMattrRGline ID::<ID> --twopassMode Basic --outSAMmultNmax 1

We used the raw junction counts from this study as the basis for DJEC DB. For this, differential junction expression analysis was implemented comparing junction abundance between each TCGA cancer type and all GTEx normal tissues. Cancer-specific changes in junction expression can be accessed through the DJE Module section in the DJEC DB web application (Supplementary Figure S2). Here, users can select target junctions to visually explore interactive splice plots and differentially expressed junctions in the context of protein domain and post-translational modifications annotated within the Prot2HG database of protein domains mapped to the human genome (Stanek et al., 2020).

In addition to RNA-seq data, the TCGA repository contains an extensive molecular and clinical annotation for tumor samples, including additional omics data (genotyping, DNA methylation, etc.) as well as multiple tumor classifications and clinical records of the patient. This data collection allows comprehensive correlation analyses between junction expression and tumor/patient traits. The junction-trait (JT) module section of DJEC DB (Supplementary Figure S3) contains significant linkages found between differentially expressed junctions and microsatellite instability (MSI) or altered oncogenic signaling pathways based on mutations, copy-number changes (CNV), mRNA expression, gene fusions and DNA methylation (Sanchez-Vega et al., 2018). This approach is an adaptation of the Matrix eQTL method (Shabalin, 2012), which uses large matrix operations of linear and ANOVA models containing covariates to account for external factors such as tumor grade or age of the patient.

Moreover, an exemplary co-expression network analysis can be also found within the JCNA section, where users can interactively explore junction expression modules as well as the results of junction-traits associations in TCGA colorectal (COADREAD) tumors (Supplementary Figure S4). This implementation of WGCNA algorithms included the removal of junctions with excessive missing values and sample outliers after sample hierarchical clustering using the *goodSamplesGenes* function (Langfelder and Horvath, 2008). The subsequent soft-thresholding procedure ensures a scale-free network, which emphasizes strong correlations between junctions and penalizes weak correlations. The scale-free network was constructed using the *blockwiseModules* function which converts the correlation matrix into a strengthened adjacency matrix that summarizes the association between all junctions.

Gene-trait correlation matrices were also calculated and used to identify and remove junctions whose correlation to external traits was gene expression-dependent. Junction co-expression modules were identified by dividing the junction expression dendrogram into branches using a dynamic tree cutting algorithm with medium sensitivity for cluster splitting (deepSplit = 2). Different colors were then assigned to the modules for subsequent visualization. MEs significance values and correlations between MEs and clinical traits were also calculated. The same was done for individual junction-to-trait correlations.

To implement cancer cell line junction expression data into DJEC DB, we downloaded fastq files from CCLE (available through the Sequence Read Archive (SRA) under accession number PRJNA523380) and carried out alignment and junction quantification with the same strategy that was previously used for TCGA and GTEx data (Kahles et al., 2018). This data was then integrated with DepMap functional genomics data in the CCLE DJE and CCLE SpliceRadar sections of DJEC DB (Supplementary Figure S5). CCLE DJE comprises the results of DJE analysis in cancer cell lines within the same tissue of origin versus fibroblasts used as “healthy” control cell lines. Significant correlations between differentially expressed junctions and gene expression, CRISPR gene effect or drug response values (DepMap 21Q3 Public, 2021) are found within CCLE SpliceRadar. Here, users can plot SpliceRadar charts with selected junction-trait associations. These database components aim to facilitate the identification of cancer cell models for specific splicing alterations and junction-trait associations that can be further studied for functional characterization in the lab.

## Results

The *DJExpress* toolbox incorporates both an R package (containing DJE and JCNA modules) and a user-friendly Shiny-based web application for a visual exploration of DJEC DB as well as custom DJE analysis for user-provided junction quantification data. Input files can either be STAR aligner-derived “SJ.out.tab” files (containing splice junction counts per sample in tab-delimited format) or any other junction quantification files as long as they contain junction IDs as first columns, following the format chr:start:end:strand (e.g., chr1:123:456:1, where positive or negative strand are coded as 1 and 2, respectively). In the following paragraphs, we describe the use of *DJExpress* and DJEC DB in detail and use case studies to demonstrate how *DJExpress* and DJEC DB can be utilized to identify and computationally explore alternative splice events across cell lines and patient samples.

### Differential Junction Expression and Junction-Trait Association Analyses in Cancer Cell Lines

To demonstrate the workflow of *DJExpress*, we analyzed cancer cell lines from the DepMap repository, comprising 13 tissue types that contain ≥30 individual cell lines per tissue (brain, breast, colon/colorectal, gastric, head and neck, kidney, leukemia, lung, lymphoma, myeloma, ovarian, pancreatic and skin cancer). Table 2 summarizes the results of DJE analysis module per tissue, using junction expression in fibroblasts as normal control condition. Users can explore this data in the DJE-CCLE section of DJEC DB.

**TABLE 2:** Summary of DJE module junction statistics in CCLE.

CCLE tissue	Quantified junctions	DE junctions	DE junctions in Group 1	DE junctions in Group 2	DE junctions in Group 3	Novel junctions	Neojunctions
Brain	120,611	846	74	73	14	3,456	110
Breast	123,349	2,153	499	431	247	3,426	255
Colon	122,639	3,363	663	722	409	3,400	336
Gastric	126,487	2,335	540	486	293	3,806	320
Head-Neck	119,194	2,398	440	391	144	3,573	316
Kidney	117,989	1,231	185	143	119	3,574	164
Leukemia	123,295	3,668	631	1,060	511	3,563	514
Lung	130,297	2,327	386	549	154	3,403	368
Lymphoma	122,911	3,795	689	1,012	524	3,772	354
Myeloma	119,528	3,307	727	678	420	3,734	398
Ovarian	122,251	1,603	295	283	238	3,512	241
Pancreatic	121,817	2,528	448	418	308	3,614	220
Skin	120,200	2,036	186	357	247	3,498	197

**FIGURE 5:**
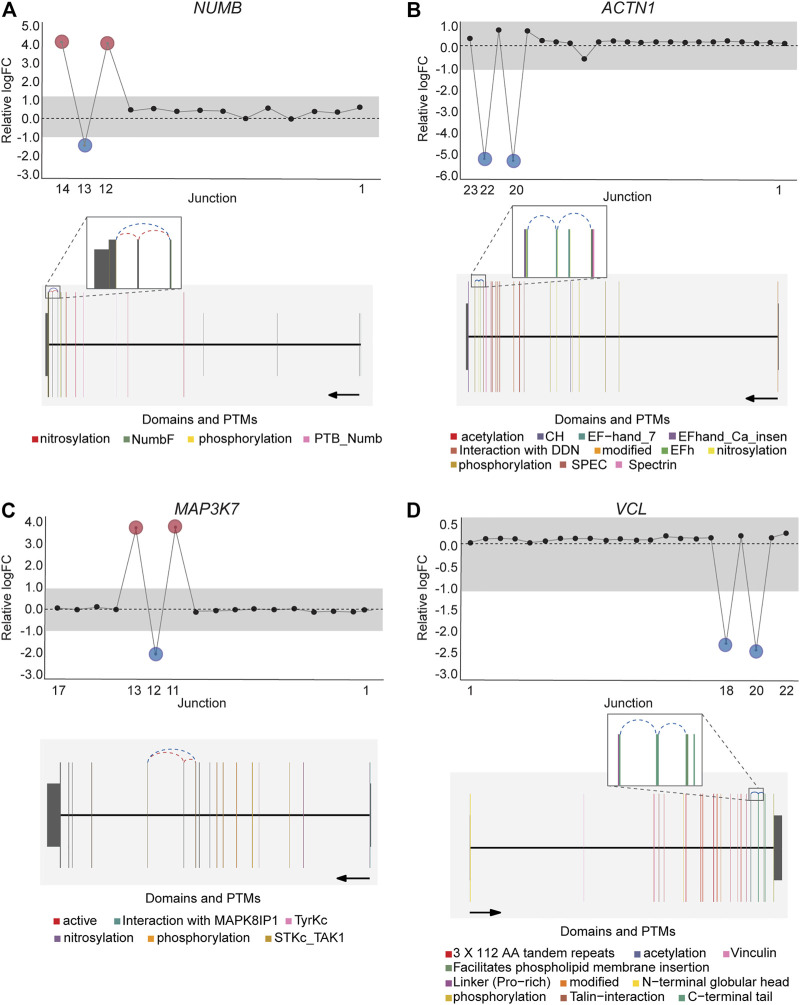
Expression profile and gene context of known alternative splicing events in cancers detected by *DJExpress* using cancer cell line data. Examples of known cancer-specific splice events are shown as gene-wise splice plots with relative logFC values (upper panels) and gene model plots with exon-to-protein domain annotation (lower panels). **(A,B)** show gene-wise splice plots of exon inclusion events in *NUMB* and *ACTN1* mRNA in breast and lung cancer cell lines, respectively. **(C,D)** show gene-wise splice plots of exon skipping events in *MAP3K7* and *VCL* mRNA in gastric and breast cancer cell lines, respectively (Numbers on the *x*-axis in the upper panels indicate the first, last and differentially used junctions in the respective gene. Grey area indicate threshold for significance (|logFC| > 1.0). Downregulated and upregulated junctions with |logFC| above threshold and significant FDR (<0.05) are shown in blue and red, respectively. These same junctions are indicated within the gene model plots as dashed arcs connecting upstream and downstream exons. Colors within exonic regions indicate the presence of protein domains and/or post translational modifications (PTMs) annotated within the Prot2HG protein domain database. Arrows below gene model plots indicate direction of transcription. Coding and UTR exons are illustrated as long and short exons respectively. Junctions with both absolute and relative logFC above the threshold (|logFC| > 1.0) but no significant FDR (>0.05) for at least one of them are shown in black).

**FIGURE 6:**
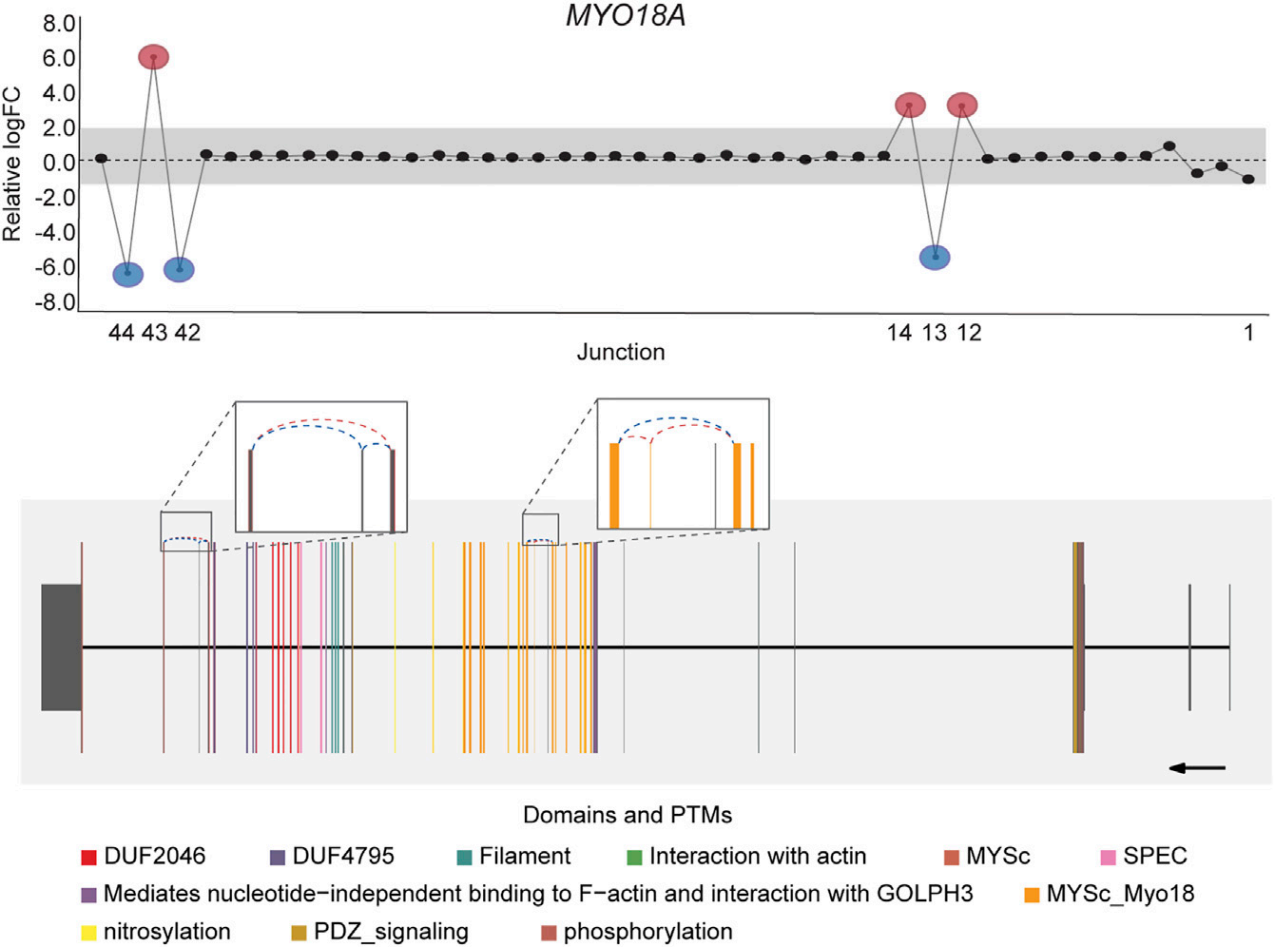
Co-regulated splicing events within *MYO18A* transcript in blood cancer. Differentially used junctions as depicted in the gene-wise splice plot in *MYO18A* indicate the concomitant inclusion of exon 11 and exclusion of exon 40 in Myeloma and Lymphoma cell lines. Gene model plot with Prot2HG-based domain annotation suggest that these co-regulated splicing events involve exonic regions containing known *MYO18A* phosphorylation sites (brown), as well as regions comprising the core myosin-like ATPase motor domain, MYSc_Myo18 (orange). *MYO18A* gene-wise splice plot in lymphoma is used as example (Numbers on the *x*-axis in the upper panels indicate the first, last and differentially used junctions in the respective gene. Grey area indicate threshold for significance (|logFC| > 1.0). Downregulated and upregulated junctions with |logFC| above threshold and significant FDR (<0.05) are shown in blue and red, respectively. These same junctions are indicated within the gene model plots as dashed arcs connecting upstream and downstream exons. Colors within exonic regions indicate the presence of protein domains and/or post translational modifications (PTMs) annotated within the Prot2HG protein domain database. Arrows below gene model plots indicate direction of transcription. Coding and UTR exons are illustrated as long and short exons respectively. Junctions with both absolute and relative logFC above the threshold (|logFC| > 1.0) but no significant FDR (>0.05) for at least one of them are shown in black).

*DJExpress* identified on average of 1,918 differentially used junctions (FDR < 0.05 and |logFC| > 1), including previously described alternative splicing events in cancer, such as the downregulation of *ACTN1* exon 19b (Gardina et al., 2006; Thorsen et al., 2008; Bielli et al., 2018), *VCL* exon 19 (Gardina et al., 2006; Thorsen et al., 2008), the upregulation of *NUMB* exon 12 (Misquitta-Ali et al., 2011; Bechara et al., 2013; Zhang et al., 2014; Zong et al., 2014), *MAP3K7* exon 12 (Munkley et al., 2019; Qiu et al., 2020; Oh et al., 2021), *CTNND1* exon 20 (Yanagisawa et al., 2008; Sebestyen et al., 2015; Wang et al., 2020), and *EXOC1* exon 11 (Ray et al., 2020; Zhang et al., 2020), as well as of exons contained within the variant domain in *CD44* (Shirure et al., 2015; Chen et al., 2018; Wang et al., 2018; Chen et al., 2020) (Figure 5; Supplementary Figure S6). Moreover, the gene-wise visualization of differential junction expression allowed the identification of complex alternative splicing patterns and isoform switches in cancer, such as the case of the co-regulated inclusion of exon 11 and exclusion of exon 40 in *MYO18A* in lymphoma and myeloma, the complex local event involving exons 15–18 in *MARK3* in leukemia, lymphoma, myeloma, breast, colon, gastric, lung and pancreatic cancer, or the isoform switches in *RGS3* in breast, colon, gastric, lung, ovarian and pancreatic cancers, and *INPP5B* in pancreatic cancer cell lines (Figure 6; Supplementary Figures S7, S8). These data demonstrate that *DJExpress* can not only reliably identify previously described alternative splicing events but can also facilitate the discovery and visualization of complex splice events within annotated splice regions.

**FIGURE 7:**
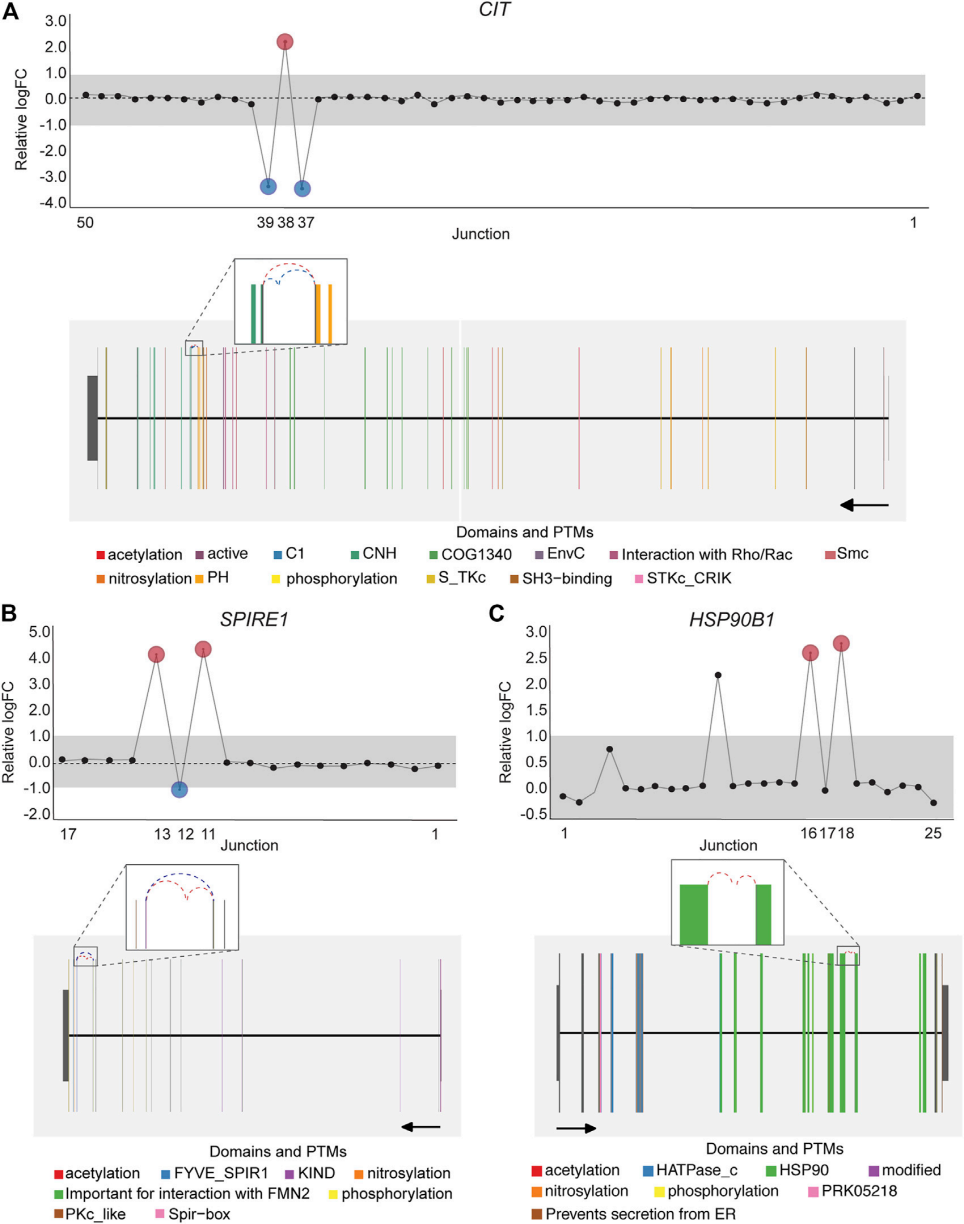
DJE analysis suggests the presence of differentially spliced non-annotated exons in cancer cell lines. Gene-wise splicing as well as gene model plots show non-annotated splice junctions whose gene location indicates the presence of exons not described in the human transcriptome annotation. **(A)** Differentially expressed non-annotated junctions between exon 37 and 38 located in the vicinity of the CNH (dark green) and PH (orange) domains in *CIT*. **(B)** Differentially expressed non-annotated junctions between exon 12 and 13 in *SPIRE1,* which contain the Spir-box domain (pink) involved in the interaction between SPIRE1 and formin (FMN)-type actin nucleators, as well as protein phosphorylation sites (yellow). **(C)** Differentially expressed non-annotated junctions between exon 13 and 14 in *HSP90B1* occurring within the HSP90 chaperone domain (green). For *CIT* and *SPIRE1* gene-wise splice plots, breast cancer is used as example. For *HSP90B1*, lung cancer is used as example (Numbers on the *x*-axis in the upper panels indicate the first, last and differentially used junctions in the respective gene. Grey area indicate threshold for significance (|logFC| > 1.0). Downregulated and upregulated junctions with |logFC| above threshold and significant FDR (<0.05) are shown in blue and red, respectively. These same junctions are indicated within the gene model plots as dashed arcs connecting upstream and downstream exons. Colors within exonic regions indicate the presence of protein domains and/or post translational modifications (PTMs) annotated within the Prot2HG protein domain database. Arrows below gene model plots indicate direction of transcription. Coding and UTR exons are illustrated as long and short exons respectively. Junctions with both absolute and relative logFC above the threshold (|logFC| > 1.0) but no significant FDR (>0.05) for at least one of them are shown in black).

Notably, an average of 3,563 non-annotated splice junctions per tissue and 292 neojunctions (defined as junctions not detected in control fibroblast cell lines) were also discovered by the DJE analysis module (Table 2). Here, the visualization of non-annotated junctions within the gene-wise DJE plots allowed us to identify the presence of previously unknown splicing events, including exon skipping, alternative 3′ splice sites, alternative 5′ splice sites and alternative first and last exons (Supplementary Figure S9). Moreover, DJE plots also revealed the presence of novel splice junctions with genomic coordinates that suggest the presence of exons so far not described in the human transcriptome annotation (Figure 7; Supplementary Figure S10). These newly identified splicing events are potentially linked to cancer physiology and their functional characterization could be subject of future studies. Nevertheless, to further illustrate the capabilities of *DJExpress* and DJEC DB, we next focused on a well-described alternative splicing switch in *NUMB* mRNA.

### Case Study 1: SpliceRadar-Based Identification of *NUMB* Alternative Splicing Regulators

*NUMB* encodes for a key determinant of cell fate that regulates the trafficking of surface proteins such as Notch, integrins and E-cadherin and can undergo alternative splicing (Nishimura and Kaibuchi, 2007; McGill et al., 2009; Teckchandani et al., 2009; Wang et al., 2009). Inclusion of *NUMB* exon 12 is frequently observed in different types of cancer, leading to a 48 amino acid extension of the proline-rich region (PRR) of the NUMB protein (Chen et al., 2009; Zhang et al., 2014; Lu et al., 2015; Rajendran et al., 2016). This longer NUMB isoform (Numb-L) was found to promote proliferation, whereas the shorter isoform (Numb-S) promotes differentiation of cancer cells (Verdi et al., 1999). In lung cancer, the splicing factor *QKI* represses the inclusion of *NUMB* alternative exon through competing with a core splicing factor SF1, thereby inhibiting proliferation and Notch signaling (Zong et al., 2014).

**FIGURE 8:**
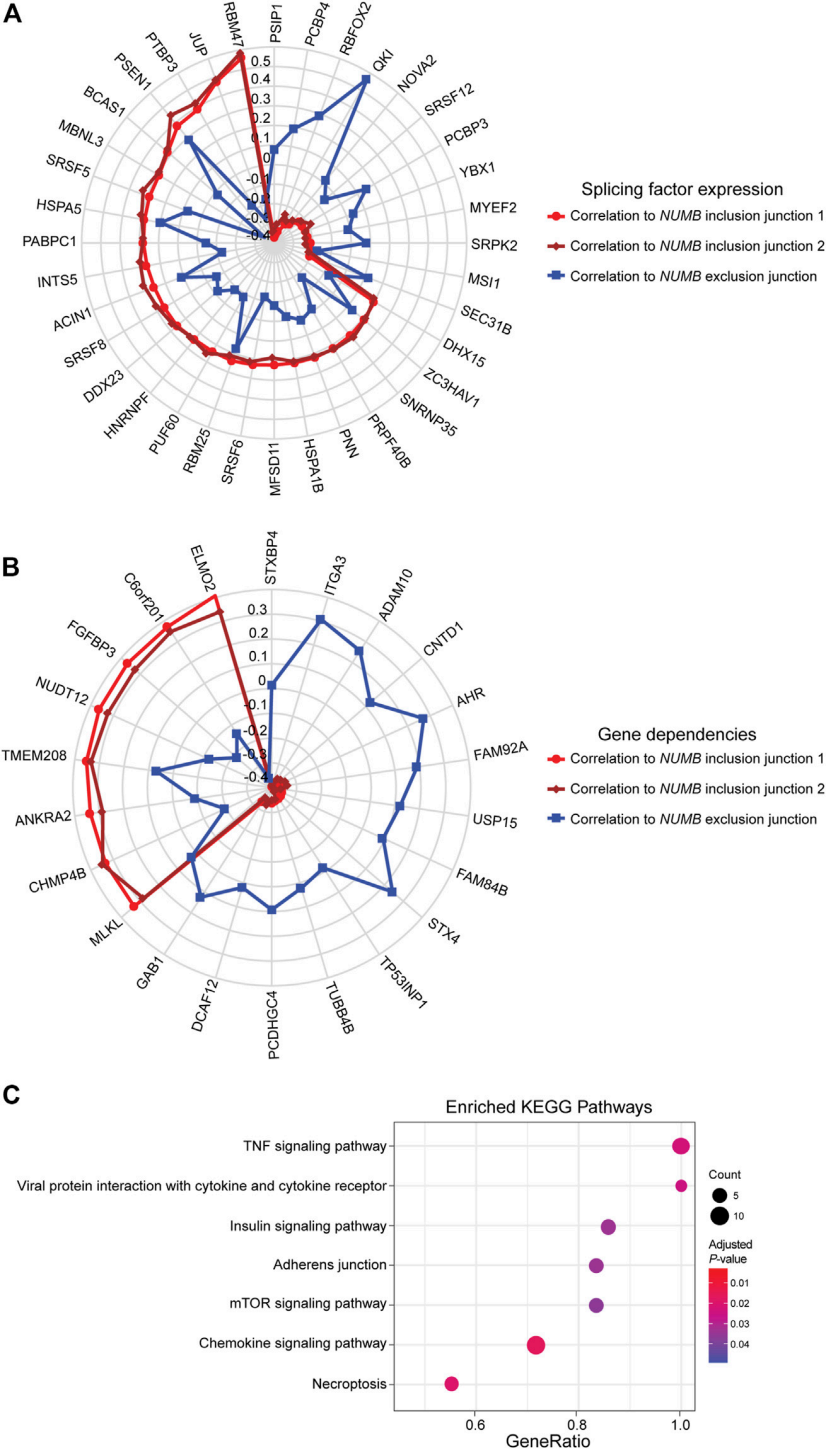
SpliceRadar plots of top trait associations to *NUMB* alternative splicing in lung cancer. **(A)** Expression of splice junctions supporting exon 12 inclusion in *NUMB* mRNA was correlated to the expression of a panel of manually curated splicing regulators in lung cancer cell lines. The top-ranked correlation coefficients (FDR < 0.05 and |rho| > 0.2) were used to construct the SpliceRadar chart with splicing factors depicted along the spokes, revealing a general trend of anti-correlation patterns to splicing factor expression between inclusion (red and dark red) and exclusion (blue) junctions. Previously known associations to *NUMB* splicing were corroborated (e.g., *QKI*, *RBFOX2* and *SRPK2*), and novel associations with similar correlation levels were identified, suggesting a more complex regulatory network of *NUMB* alternative splicing than previously described. **(B)** SpliceRadar plot showing top-ranked correlations (FDR < 0.05 and |rho| > 0.2) between exon inclusion junction expression in *NUMB* and gene dependencies (defined as gene loss effect on cell survival) using DepMap CRISPR screen data. Anti-correlation patterns of dependency values and expression of inclusion and exclusion junctions are also observed as in the case of panel **(A)**. **(C)** KEGG pathway enrichment analysis using gene names of significantly associated dependencies ranked by correlation coefficient. The enrichment plot shows top over-represented pathways within *NUMB* splicing-correlated gene dependencies (Dot size represents the number of genes in each KEGG pathway, color gradient indicates significance level of adjusted *p*-values).

This well-documented *NUMB* isoform switch was also detected with *DJExpress*, which showed a ∼16-fold (log_2_ ∼4-fold) upregulation of *NUMB* exon 12 inclusion junctions in breast cancer cell lines compared to fibroblasts (Figure 5A). A similar *NUMB* splice pattern was observed across other cancer types (data not shown). Furthermore, by using *DJExpress* JT module, we corroborated the positive correlation between *QKI* gene expression and *NUMB* exon 12 exclusion (Figure 8A). Moreover, SpliceRadar-based visualization identified additional positively and negatively correlated splicing regulators, including *SRPK2* and *RBFOX2*, which have both previously been implicated in the regulation of *NUMB* alternative splicing (Lu et al., 2015). Thus, our data suggests that the control of *NUMB* alternative splicing in cancer may involve a more complex regulatory network than previously thought. These data demonstrate that *DJExpress* can not only validate known associations with splice events but can also, through functionality of the SpliceRadar tool, identify additional regulatory networks that may be altered in cancer.

DJEC DB incorporates gene dependencies and drug response data from the DepMap repository. We thus expanded the landscape of phenotypic associations to *NUMB* alternative splicing in lung cancer cell lines (Figure 8B). Pathway enrichment analysis of significantly associated gene dependencies revealed enrichment of components within the mTOR and insulin signaling pathways. This is consistent with previous studies, which suggested that activated ERK signaling is a common mechanism that regulates *NUMB* isoform expression in breast and lung cancer cells (Rajendran et al., 2016) (Figure 8C). Similarly, SpliceRadar plots using top correlations with drug response values also revealed associations between the expression of exon-inclusion junctions in *NUMB* and cell survival rates after treatment with several compounds targeting PI3K/mTOR and ERK MAPK signaling (Supplementary Figure S11). These data reinforce the notion of a functional connection between *NUMB* exon 12 inclusion and pro-inflammatory signaling cascades.

Taken together, these results illustrate the potential of the *DJExpress* pipeline to identify *bona fide* differentially expressed splice junctions and reveal physiologically relevant associations between junction expression and various external traits. Thus, *DJExpress* can be used to support and generate hypotheses regarding the potential molecular mechanisms involved in the regulation and physiological consequences of alternative splicing.

### DJEC DB Data Summary

TCGA project is a large-scale oncology study that has allowed the comprehensive characterization of multiple cancer types using a catalogue of clinical and molecular data, including RNA sequencing from thousands of patients across multiple tumor types. This resource harbors an excellent opportunity for cancer researchers and clinicians to explore and define tumor-specific transcriptomic signatures, and to integrate them with additional external traits such as mutations, copy number variations (CNV) or microsatellite instability (MSI). These features of TCGA can facilitate identification of novel therapeutic or diagnostic biomarkers. However, TCGA alternative splicing analyses, particularly the association of splice events with clinical and molecular traits, is currently not available in an accessible way.

To fill this gap, we generated DJEC DB, a platform that provides an integration of differential junction expression analysis with TCGA molecular and clinical data. For this, we used splice junction quantification from a recently published study (Kahles et al., 2018) where TCGA and GTEx RNA-seq samples were re-analyzed using 2-pass STAR alignment, thereby allowing identification of annotated and *de novo* splice events. Additionally, we quantified junction expression in cancer cell lines from CCLE fastq files and integrated this data with functional genomics data sets from the DepMap repository.

DJEC DB comprises four main sections: 1) Differential Junction Expression (DJE) in TCGA vs GTEx tissue, 2) Junction-Trait (JT) associations using external clinical and molecular sample data, 3) Junction Co-expression Network Analysis (JCNA) using junction expression in colorectal (COADREAD) tissue samples as example dataset, and 4) Differential Junction Expression in cancer cell lines and association with DepMap functional genomics data (DJE-CCLE).

The DJE section comprises summary statistics and visualization options for an average of 6,345 differentially expressed junctions across the 32 tumor tissue types analyzed (FDR <0.05 and |logFC| > 2, Table 3). In the JT section, an average of 674 statistically significant associations are shown between differentially expressed junctions and altered oncogenic signaling pathways determined by the presence of mutations, CNVs, altered gene expression, gene fusions, DNA methylation and MSI (in the case of COADREAD tumors).

**TABLE 3:** Summary of DJE and JT junction statistics in DJEC DB.

TCGA tissue cohort	Sample size	Quantified junctions	DE junctions	Associations to genomic alterations	Associations to mutations	Associations to pathway alterations
ACC	79	13,827,029	2,335	1	2	—
BLCA	408	14,369,479	2,935	215	274	—
BRCA	1,083	15,445,200	3,740	334	306	15
CESC	304	14,260,819	4,808	14	20	—
CHOL	36	13,786,637	8,446	10	10	—
COADREAD	372	14,315,224	5,534	49	44	—
DLBC	48	13,822,896	6,150	9	5	—
GBM	165	13,995,214	12,781	2	4	—
HNSC	500	14,592,967	5,745	49	117	2
KIPAN	738	14,965,143	2,836	92	93	1
LGG	526	14,536,867	6,771	6,708	6,061	404
LIHC	372	855,905	4,996	97	99	—
LUAD	516	14,681,817	3,931	153	149	—
LUSC	500	14,804,638	4,721	107	114	10
MESO	82	13,866,293	4,078	—	—	—
OV	199	16,204,728	8,509	9	10	—
PAAD	178	13,981,645	4,942	26	26	—
PCPG	183	14,428,362	8,973	228	228	—
PRAD	497	1,166,561	4,097	85	94	—
SARC	257	14,106,882	1,810	12	50	—
SKCM	471	14,106,882	3,436	16	11	—
STES	535	18,214,111	7,155	418	330	—
TGCT	156	14,050,087	9,684	14	14	—
THCA	500	14,437,693	4,885	699	714	37
THYM	118	13,939,486	3,860	30	31	—
UCEC	179	14,038,958	9,241	114	99	—
UCS	56	13,829,412	9,091	6	5	—
UVM	80	13,809,902	9,285	—	—	—

To exemplify the use of the JCNA approach, we selected the 372 samples from the TCGA COADREAD tumor cohort to construct a junction co-expression network (see methods for details). For this, we used a minimum module size of 20 junctions and an unsigned network type, meaning that the weight of connection between nodes (junctions) is calculated irrespectively of the direction of the association, so modules can contain both, positively and negatively correlated junctions (Supplementary Figure S4).

From a total of 7,404 junctions filtered by their gene expression-independent association to sample traits, 36 expression modules were found for this tumor type, with an average of 206 junctions per module. Module-trait associations were also determined throughout the correlation between ME expression values and tumor stage, MSI, mutations in TP53, EGFR, KRAS and BRAF genes, as well as expression across six splicing factor gene modules previously calculated from gene expression data.

Finally, the DJE-CCLE section contains the results of the differential junction expression analysis of normal fibroblast cells vs cancer cell lines clustered by tissue of origin, as described above. Significant correlations between junction expression and functional genomics data obtained from the DepMap repository are displayed in a summary table and selected association patterns can be visualized using SpliceRadar plots.

### Search and Browse DJEC DB

Within the DJE section, users can first define the target tumor tissue type as well as the logFC and FDR cutoffs for the significance in differential expression (Supplementary Figure S2). A table with the summary statistics is displayed and specific target genes or junctions can be selected by the users in order to display gene-wise splice plots as well as a zoomable gene model plots with exon-to-protein domain annotation. In addition, junction-trait associations in TCGA can be explored within the JT section following user-defined tumor tissue type and external molecular trait options (Supplementary Figure S3).

For the JCNA section using the TCGA COADREAD sample cohort, a junction dendrogram with expression module assignment, as well as a module-trait association heatmap are displayed (Supplementary Figure S4). For intramodular analysis, users can select specific modules and traits to visualize module-to-trait significance plots, as well as module networks in interactive format. Both are helpful in identifying centrally located intramodular hub junctions with high module membership as well as high significance for selected traits. This allows the user to generate testable hypotheses about junction module expression, regulation and association to cancer phenotypes that can be implemented in validation experiments.

Similar interactive visualization can be also found within the DJE-CCLE section. Here, users can select the tissue of origin, the significance cutoff for differential expression, as well as target genes/junctions and junction-trait associations to be displayed in gene-wise splice and SpliceRadar plots (Supplementary Figure S5).

### Case Study 2: Cancer Cell Line DJE Signature Is Recapitulated by Tumor Tissue Analysis in DJEC DB

**FIGURE 9:**
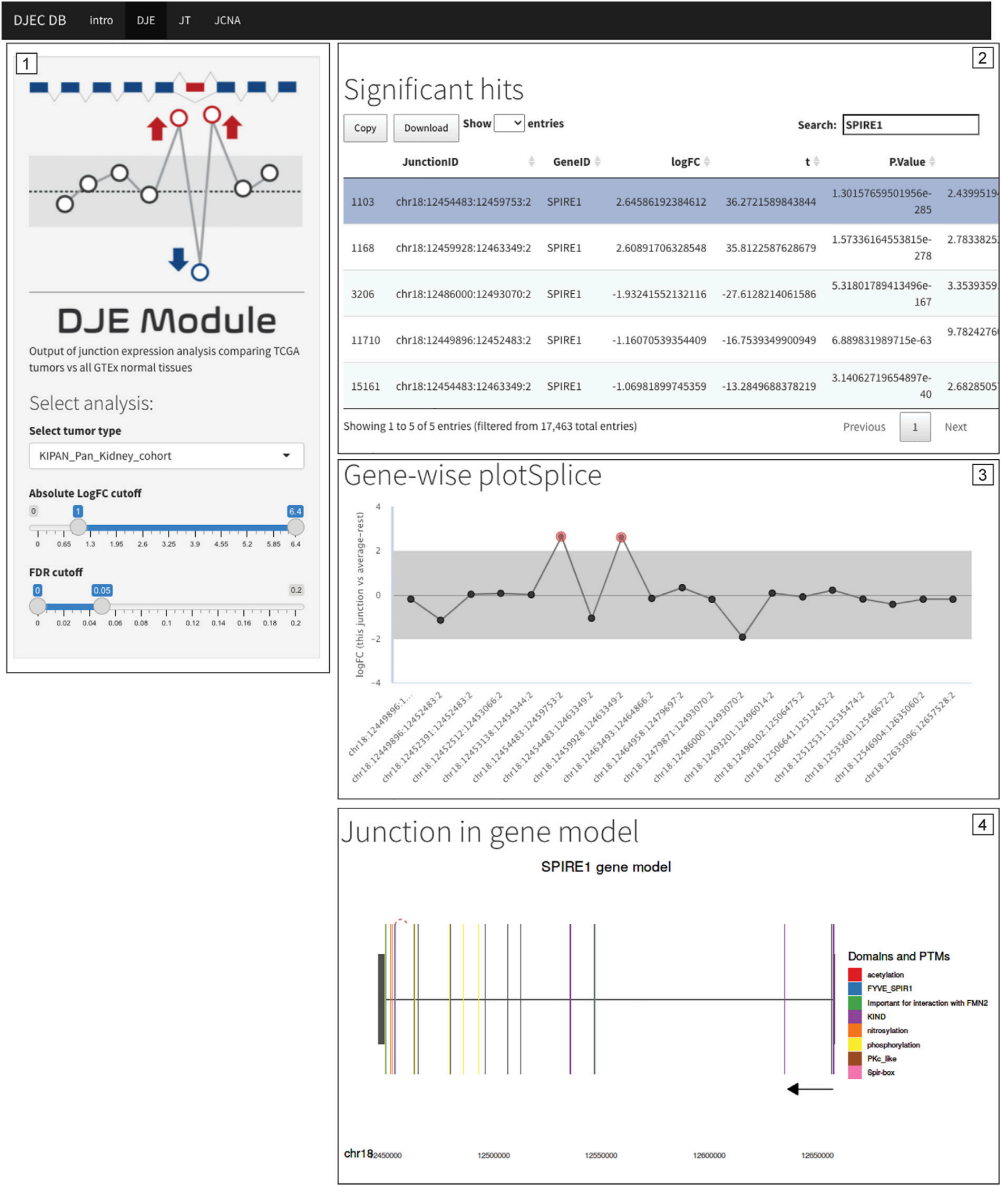
Differentially expressed non-annotated junctions in *SPIRE1* are also found in the context of primary tumor tissue. Differential expression of junctions suggesting the presence of a non-annotated exon in *SPIRE1* mRNA were not only identified in cancer cell lines (see Figure 7B) but are also found in BRCA, LUAD, KIPAN, PRAD, and THCA TCGA cohorts. Caption of DJEC DB DJE analysis in KIPAN is shown as example. The exon inclusion event can be found by filtering for differentially expressed junctions following cutoff criteria of <0.05 for FDR and |logFC|>1.0 (Panel 1) and then selecting any of the two inclusion junctions based on their genomic coordinates (Panel 2). DJEC DB displays gene-wise splice plots (Panel 3) as well as domain-annotated gene model plots (Panel 4).

One of the central features of DJEC DB is the possibility to interrogate the presence of alternative splicing patterns observed in cancer cell lines in the context of tumor tissues. *NUMB*, *VCL*, *MAP3K7* and *EXOC1* exon skipping events are examples of known splicing events that can be also observed in tumor tissue (Supplementary Figures S12–S15). Notably, the presence of a differentially expressed non-annotated exon between exon 12 and 13 in *SPIRE1*, which we detected in cancer cell lines (Figure 7B), was also identified in BRCA, LUAD, KIPAN, PRAD, and THCA cohorts by DJEC DB data using gene-wise splicing visualization (Figure 9). This suggests that the alternative inclusion of this previously unknown region in *SPIRE1* transcript may be a common feature across different cancer types *in vitro* and *in vivo*. These data demonstrate the applicability of DJEC DB in identifying and cross-validating potentially oncogenic alternative splicing patterns both in cancer cell lines and tumor tissue.

The JT module in DJEC DB provides a workflow to associate junction expression with user-provided molecular or clinical traits. In the case of *CTNND1* splicing event, we found significant associations between the expression of exon 20 inclusion junctions and *TP53* mutation status in BRCA, as well as with amplification of *CCND1* gene and epigenetic silencing of *CDKN2A* in STES (Supplementary Figure S16). This is consistent with previous studies indicating that *CCND1* isoforms expression regulates cell proliferation and cell cycle progression by controlling the levels of cyclin proteins in cancer cells (Chartier et al., 2007; Jiang et al., 2012; Liu et al., 2014).

Taken together, these data corroborate DJEC DB as a valuable bioinformatics resource for the exploration and visualization of differential junction expression, as well as for the interrogation of physiologically relevant junction-trait associations in the context of global splicing analysis in cancer cell lines and tumor tissue.

## Discussion

With the increasing availability of NGS data sets, the possibility to perform transcriptome-wide alternative splicing analysis has become a commonality rather than an exception in disease research. Nevertheless, computational analysis pipelines that allow the broad research community to effortlessly interrogate alternative splicing phenotypes are largely missing.

Our custom pipeline, *DJExpress*, aims to address this issue. With *DJExpress*, we have incorporated multiple existing algorithms in a novel computational approach for differential splicing analysis, which is suitable for analysis of small-scale as well as large-scale splice junction datasets. Moreover, *DJExpress* allows the analysis of millions of exon-exon boundaries per sample, using *limma’s* statistical framework. *Limma’s* algorithm has been shown to be highly accurate for gene expression analysis (Law et al., 2014; Corchete et al., 2020; Gerard, 2020), although a comprehesive analysis of accuracy for splicing is beyond the scope of this work and remains as a future direction. Nevertheless, the implication of *limma* methodology proved to be highly flexible. This is not only the case in terms of model specification (any contrast in a linear model including the use of continuous as well as categorical predictors can be related to differential junction expression) but also for the various parameters introduced into the fit model, including posterior variance estimators, observation weights and variance modelling. These features, together with *limma’s* additional data pre-processing methods such as variance stabilization, all help to improve inference of differential junction expression.

Importantly and similar to gene expression studies (Peixoto et al., 2015), removing or accounting for both known and unknown confounding factors (e.g., technical biases such as batch effects, or population structure such as molecular or clinical subtypes) is crucial when analyzing alternative splicing phenotypes in RNA-Seq data sets (Slaff et al., 2021). Confounding factors can greatly increase the numbers of false positives and negatives, which ultimately will affect interpretation of potential biological relationships. Thus users should test for potential known confounder effects in their data, for example by using PCA or UMAP plots, and use dedicated tools to correct for confounders such as limma, ComBat, RUV, SVA and MOCCASIN (Leek, 2014; Risso et al., 2014; Zhang et al., 2020; Slaff et al., 2021).

Apart from these statistical aspects, *DJExpress* provides a comprehensive framework to graphically summarize differential splicing. The adapted *limma*-based visualization approach allows inspection of alternative splicing not only at the level of individual junction loci, but also in the presence of more complex splicing patterns. These can involve simultaneous changes in the expression of multiple junctions across the entire gene. This is particularly advantageous, considering that existing splicing analysis tools are either focused on the definition of local alternative splicing events which can be both simple (exon skipping, alternative 3′ or 5′ splice sites, etc.) or complex (simultaneous occurrence of multiple splice events in a given mRNA), or only allow detection of known transcript isoforms. Thus, most previous tools disregard the simultaneous visual representation of the full spectrum of up- and down-regulated splicing patterns in a gene that is retrieved through junction quantification. Broadly used exceptions are LeafCutter (Li et al., 2018) and MAJIQ (Vaquero-Garcia et al., 2016), which can both also represent complex splicing changes across the entire mRNA.

Notably, the differential junction usage analysis by *DJExpress* does not allow a direct assessment of intron retention events, which require intron and intron-exon junction read counts for their quantification. Nevertheless, dedicated tools such as MAJIQ (Vaquero-Garcia et al., 2016), IRFinder (Middleton et al., 2017), iREAD (Li et al., 2020) or S-IRFinder (Broseus and Ritchie, 2020) are specifically designed for quantification of intron retention events and are thus well-suited for this specific type of analysis.

Recently, RNA-seq data from TCGA and GTEx was integrated within a large transcriptomic profiling workflow, including splicing quantification of more than 20,000 human normal and tumor tissue samples (Kahles et al., 2018). Although this study provided unified splicing data across healthy and tumor tissue, the analysis is based on the construction of complex splicing graphs across thousands of samples and genes which are difficult to access and interpret. Furthermore, approaches to explore the data in a graphically visualized format were not the scope of this previous study. This limited the availability and accessibility of this data for the general research community as well as the feasibility of splicing-trait association analyses using genomic, epigenetic, and clinical records available within the TCGA repository. These points are addressed by *DJExpress* and DJEC DB which facilitate easy access, analysis and visualization of cancer splicing data. Moreover, by providing a simple analysis workflow for custom data sets, our pipeline is not restricted to cancer researchers but can be used to pursue a broad variety of alternative splicing-related scientific questions.

In conjunction with the usability of the *DJExpress* for differential splicing analysis and visualization using custom RNA-Seq data, the multidimensional integration of cancer data within DJEC DB represents a comprehensive resource of cancer-specific splicing signatures and junction-trait associations. We demonstrated that our pipeline has the potential to unveil novel splicing-related molecular signatures, which may contribute to improved patient stratification and more effective cancer treatment strategies. Moreover, the integration of DepMap data allows association of junction expression with molecular features such as gene dependencies and drug response profiles. This will help researchers to identify cancer cell models for specific splicing alterations that can then be used for functional characterization in the lab.

Another recently established cancer splicing repository, RJunBase (Li et al., 2021), follows a similar splicing analysis strategy as DJEC DB. While focusing on back-splice and fusion junctions, RJunBase provides splicing patterns at junction level and median junction expression information in GTEx and TCGA samples. However, it lacks differential junction expression analyses between cancer and healthy tissue and does not include association of splice events with molecular or clinical data. Thus, compared to RJunBase, DJEC DB not only includes differential junction expression analyses but also provides functional associations of splicing changes with phenotypic traits. These features make DJEC DB a comprehensive data base that can facilitate the discovery of novel cancer-related aberrant splicing patterns with potential phenotypic consequences.

Taken together, *DJExpress* provides researchers with a comprehensive toolbox for exploration of alternative splicing phenotypes in health and disease, and, with DJEC DB, includes multi-level data of alternative splicing signatures in healthy tissue, tumors and cancer cell lines.

## Data Availability

GTEx and TCGA raw junction counts were provided by Dr. Andre Kahles (Biomedical Informatics Group, Department of Computer Science, ETH Zürich). All TCGA molecular and clinical data sets used in this study are publicly available and can be found here: https://portal.gdc.cancer.gov/. All cell line functional genomics data used in this study is publicly available and can be found here: https://depmap.org/portal/download/. All raw RNA-Seq data files of cell lines from CCLE are available through the Sequence Read Archive under accession number PRJNA523380. All additional data and code are available from the authors upon reasonable request. *DJExpress* R package is available at https://github.com/MauerLab/DJExpress. DJEC DB database is available at https://gitlab.com/mauerlabrsc/djecdb.

## Footnotes

1
https://depmap.org/.

## Supplementary Material

Supplementary Figure 1 Performance evaluation of DJE module. Median **(A)** and log2 median **(B)** process time following 10 repetitions of data import (*DJEimport*), junction annotation (*DJEannotate*), expression filtering (*DJEprepare*), normalization and differential junction expression analysis (*DJEanalyze*) within the DJE module of *DJExpress.* **(C)** Median memory consumption (in bytes) of the entire DJE module. Error bars represent standard deviations. Default settings with increasing sample size and random relative group sizes are used in the analysis.

Supplementary Figure 2 Interactive DJE visualization in tumors using DJEC DB. **(A)** Start interface of the DJE section in DJEC DB. Panel 1 highlights the selection option section. Users can define the TCGA tumor type, and the significance cutoff for differential junction usage based on minimal |logFC| and FDR values. Panel 2 shows the downloadable summary statistics table for junctions passing the selected cutoff. Here, users can filter junctions by browsing specific gene IDs, junction IDs or genomic coordinates. After selecting a target junction by clicking over it on the table, gene-wise splice plots as well as junction in domain-annotated gene model context (Panels 3 and 4 respectively) can be interactively visualized. Hovering over each junction in the gene-wise splice plot displays a box with summarized DJE information, including relative and absolute logFC values, FDR values and expression group of the selected junction. Colors within exonic regions in the gene model plot indicate the presence of protein domains and/or post-translational modifications (PTMs). The position of the selected junction within the gene model plot is indicated by a dashed arc whose color correspond to the type of differential expression (blue for downregulation and red for upregulation). Specific regions within the gene model plot (e.g., position of the selected junction) can be further explored by cursor selection, which displays a zoomed image version of the selected gene region. **(B)** KIF13A exon inclusion event in BRCA TCGA cohort is used as an example. Significance cutoff was set to |logFC| > 2.0 and minimal FDR cutoff of 0.05. The two exon inclusion junctions are shown in red within the gene-wise splice plot, and the gene model plot indicate the position of the selected junction, which happens close to an annotated phosphorylation site of the protein.

Supplementary Figure 3 Visualization of JT section within DJEC DB. This section contains the results of the junction-trait association analyses using ANOVA and linear models from *Matrix eQTL* methods (Shabalin, 2012). Differentially expressed junctions within each TCGA tumor type were associated to microsatellite instability (MSI) or altered oncogenic signaling pathways based on mutations, copy-number changes (CNV), mRNA expression, gene fusions and DNA methylation (Sanchez-Vega et al., 2018). Users can select the tissue of interest, as well as the trait to which junction expression is associated (Panel 1). A downloadable summary statistics table is displayed (Panel 2), where specific genes, junctions, genomic coordinates or traits can be browsed. When a specific association is selected from the table, interactive junction-trait association boxplots are displayed (Panel 3) and hoovering over them shows summarized statistics of the analysis. The image contains the example of the association between a differentially expressed junction in the transcript of S100 Calcium Binding Protein A14 (*S100A14)* and MSI, with high levels of MSI (MSI-H) in tumors (violet) being associated to significantly more inclusion levels of the junction than low levels of MSI (MSI-L) (red) and microsatellite stable (MSS) (blue) colorectal tumors.

Supplementary Figure 4 Junction Co-expression Network Analysis (JCNA) of TCGA COADREAD in DJEC DB. **(A)** JCNA section comprises the results of the junction co-expression analysis across the 372 samples from the TCGA COADREAD tumor type. 7,404 junctions where clustered into 36 expression modules. The dendrogram of clustered junctions is displayed (panel 2), where each branch in the figure represents one junction, and every color below represents one co-expression module. The heatmap of module-trait associations (panel 3) based on correlation coefficients between junction modules and traits is also shown (blue and red indicate positive and negative correlations respectively). Traits are in the x-axis and junction modules with their respective assigned letter and color are in the y-axis. Traits analyzed include Microsatellite instability (MSI), BRAF, KRAS EGFR and TP53 mutation status, tumor stage and 6 co-expression modules of splicing factors calculated for COADREAD samples (SFG1-6). **(B)** Interactive scatter diagram of module membership vs. junction significance is shown when users select specific traits and modules within the selection options section (panel 1). **(C)** For the selected module, an interactive junction network is also displayed. Each node in the network represents a single junction. Junctions are colored based on gene ID. Users can select target genes within the network to highlight their respective junctions (e.g., EDEM2 junctions in the zoomed image).

Supplementary Figure 5 Visualization of junction-trait associations using DepMap gene dependencies within JT-CCLE section in DJEC DB. This section contains the results of the junction-trait correlation analyses using junction expression and genome-wide gene dependency screens in cancer cell lines. Users can select the tissue of interest, as well as the absolute correlation coefficient cutoff to be used for SpliceRadar visualization (panel 1). A downloadable correlation matrix is displayed (panel 2), where specific genes, junctions, genomic coordinates or traits can be browsed. When specific junctions are selected (maximum 3) from the table, interactive SplicePlots with top 50 junction-dependencies correlations are displayed (panel 3). An example of significant associations between *MYO18A* exon 40 expression and gene dependencies in lymphoma cell lines is shown.

Supplementary Figure 6 Illustration of known alternative splicing in cancer using DJEC DB. **(A)** Cancer-specific inclusion of exon 11 in *EXOC1* involving differentially used junctions 11, 12 and 13. The alternative splicing events occurs within the C-terminus Sec3_C domain (pink) and adjacent to several phosphorylation sites (brown) as depicted by the domain-annotated gene model plot. **(B)** Exon 20 inclusion event in *CTNND1*, involving junctions 20 and 23. This exon localizes at the C-terminal domain of *CTNND1* and in the vicinity of several phosphorylation sites as indicated in the gene model plot. **(C)** Differentially used junctions are depicted within the gene-wise splice plot in *CD44* (downregulated junction indicating the exclusion of the variable region and upregulated junctions indicating the inclusion of exons 7–14 within the variable region). Gene model plot with Prot2HG-based domain annotation indicate that the variable region in *CD44* correspond to the proteolytically cleavable extracellular Stem domain (dark gold) as previously described. For differential junction expression in *EXOC1*, *CTNND1* and *CD44*, colon, pancreatic and breast cancer cell line are shown as examples, respectively. (Numbers on the x-axis in the upper panels indicate the first, last and differentially used junctions in the respective gene. Grey area indicate threshold for significance (|logFC| > 1.0). Downregulated and upregulated junctions with |logFC| above threshold and significant FDR (< 0.05) are shown in blue and red, respectively. These same junctions are indicated within the gene model plots as dashed arcs connecting upstream and downstream exons. Colors within exonic regions indicate the presence of protein domains and/or post translational modifications (PTMs) annotated within the Prot2HG protein domain database. Arrows below gene model plots indicate direction of transcription. Coding and UTR exons are illustrated as long and short exons respectively. Junctions with both absolute and relative logFC above the threshold (|logFC| > 1.0) but no significant FDR (> 0.05) for at least one of them are shown in black. Junctions with either relative or absolute logFC below the indicated threshold are shown in grey).

Supplementary Figure 7 Example local complex event in *MARK3* transcript in several cancer types. **(A)** Differentially used junctions as depicted in the gene-wise splice plot and gene model plot in *MARK3* indicate the presence of a splicing event involving several co-regulated junctions between exons 15–18 (the event accounts for a double exon skipping event, where several exon-exon junctions, including an alternative 3′ splice site event are downregulated). CCLE Breast cancer vs fibroblast analysis cell lines is used as example. (Numbers on the x-axis in the upper panels indicate the first, last and differentially used junctions in the respective gene. Grey area indicate threshold for significance (|logFC| > 1.0). Downregulated and upregulated junctions with |logFC| above threshold and significant FDR (<0.05) are shown in blue and red, respectively. These same junctions are indicated within the gene model plots as dashed arcs connecting upstream and downstream exons. Colors within exonic regions indicate the presence of protein domains and/or post translational modifications (PTMs) annotated within the Prot2HG protein domain database. Arrows below gene model plots indicate direction of transcription. Coding and UTR exons are illustrated as long and short exons respectively. Junctions with both absolute and relative logFC above the threshold (|logFC| > 1.0) but no significant FDR (>0.05) for at least one of them are shown in black). **(B)** *DJEplotSplice* function in *DJExpress* allows the alternative interactive visualization of all found junctions for a target gene within the original junction quantification data, including those removed after coverage filtering. The full gene-wise plot of *MARK3* reveals the presence of 1084 junctions detected across all analyzed samples. Junctions filtered out for differential analysis based on user-defined expression cutoffs are shown in clear grey. *DJEplotSplice* output offers an additional read coverage information across the gene using the loess fit of median junction read count (blue line) as readout. Numbers in the x-axis of the read coverage plot indicate genomic coordinates of *MARK3* gene structure.

Supplementary Figure 8 Examples of isoform switches detected by *DJExpress* in cancer cell lines. Visualization of differentially used junctions within gene-wise splice plots and gene model plots reveals cases of upregulation and downregulation of specific transcript isoforms. **(A)** *INPP5B* gene-wise splice plot in pancreatic cancer cell lines indicates the presence of one upregulated junction and a series of consecutive downregulated junctions at the 5′ region of the gene. When compared to the transcript isoform annotation for *INPP5B*, this pattern is indicative of downregulation of the long *INPP5B* isoform (bottom right) containing five additional exons at the 5′ region which corresponds to the Type II inositol 1,4,5-trisphosphate 5-phosphatase PH protein domain (INPP5B_PH) (green), while the short isoform (top right) containing an alternative first exon downstream of the INPP5B_PH domain appears upregulated. **(B)** *RGS3* isoform switch is also observed in breast, colon, gastric, lung, ovarian and pancreatic cancers. The series of upregulated junctions belongs to a long isoform version of *RGS3*, while downregulated junctions correspond to a shorter transcript variant with an alternative downstream promoter. This short isoform shares its second and third exon with the long isoform but differs in four downstream exons containing the Regulator of G protein Signaling (RGS_RGS3) (brown) protein domain. *RGS3* gene-wise splice plot in gastric cell lines is shown as example (Numbers on the *x*-axis in the upper panels indicate the first, last and differentially used junctions in the respective gene. Grey area indicate threshold for significance (|logFC| > 1.0). Downregulated and upregulated junctions with |logFC| above threshold and significant FDR (<0.05) are shown in blue and red, respectively. These same junctions are indicated within the gene model plots as dashed arcs connecting upstream and downstream exons. Colors within exonic regions indicate the presence of protein domains and/or post translational modifications (PTMs) annotated within the Prot2HG protein domain database. Arrows below gene model plots indicate direction of transcription. Coding and UTR exons are illustrated as long and short exons respectively. Junctions with both absolute and relative logFC above the threshold (|logFC| > 1.0) but no significant FDR (>0.05) for at least one of them are shown in black. Junctions with either relative or absolute logFC below the indicated threshold are shown in grey).

Supplementary Figure 9 Example of alternative splicing event types identified by *DJExpress*. Differentially used non-annotated junctions are representative of different types of alternative splicing events. **(A)** *XRCC6* gene-wise splice plot in breast cancer cell lines indicates the presence of an alternative 3′ splice site (A3’SS) in exon 6. This event occurs within the Von Willebrand factor type A protein domain (vWA_ku) (pink) known to be involved in protein-protein interactions. **(B)** An alternative first exon (AFE) event is detected in *BIN1* in lymphoma cell lines. The downregulated first exon is known to contain a region required for interaction with *BIN2* (orange). **(C)** Detection of an alternative 5′ splice site (A5′SS) involving the first exon of *LDLRAP1* in myeloma. **(D)** The upregulated junction in *C11orf58* in brain cancer cell lines indicates the presence of both, an alternative 5′ splice site (A5′SS) and an alternative 3′ splice site (A3′SS) in exon 2 and 3, respectively, which occurs inside the region corresponding to the Small acidic protein family (SAMP) domain (pink) (Numbers on the *x*-axis in the upper panels indicate the first, last and differentially used junctions in the respective gene. Grey area indicate threshold for significance (|logFC| > 1.0). Downregulated and upregulated junctions with |logFC| above threshold and significant FDR (<0.05) are shown in blue and red, respectively. These same junctions are indicated within the gene model plots as dashed arcs connecting upstream and downstream exons. Colors within exonic regions indicate the presence of protein domains and/or post translational modifications (PTMs) annotated within the Prot2HG protein domain database. Arrows below gene model plots indicate direction of transcription. Coding and UTR exons are illustrated as long and short exons respectively. Junctions with both absolute and relative logFC above the threshold (|logFC| > 1.0) but no significant FDR (>0.05) for at least one of them are shown in black).

Supplementary Figure 10 Example of a differentially spliced non-annotated exon in cancer cell lines. Differentially expressed non-annotated junctions indicate the presence of an exon inclusion event (junctions 18–20) between exon 17 and 18 involving the actin-binding module (I_LWEQ) (violet) in *TLN1* as observed in the domain-annotated gene model plot. *TLN1* plots in breast cancer cell lines are used as example (Numbers on the *x*-axis in the upper panels indicate the first, last and differentially used junctions in the respective gene. Grey area indicate threshold for significance (|logFC| > 1.0). Downregulated and upregulated junctions with |logFC| above threshold and significant FDR (<0.05) are shown in blue and red, respectively. These same junctions are indicated within the gene model plots as dashed arcs connecting upstream and downstream exons. Colors within exonic regions indicate the presence of protein domains and/or post translational modifications (PTMs) annotated within the Prot2HG protein domain database. Arrows below gene model plots indicate direction of transcription. Coding and UTR exons are illustrated as long and short exons respectively. Junctions with both absolute and relative logFC above the threshold (|logFC| > 1.0) but no significant FDR (>0.05) for at least one of them are shown in black).

Supplementary Figure 11 SpliceRadar plot of top associations between *NUMB* alternative splicing and drug treatment response in lung cancer. Expression of splice junctions involved in the exon inclusion event of *NUMB* was correlated to cell survival rates after drug treatment using DepMap drug screens data in lung cancer cell lines. The top-ranked correlation coefficients (FDR < 0.05 and |rho| > 0.2) were used to construct the SpliceRadar plot. A general trend of anti-correlation patterns with inclusion (red and dark red) and exclusion (blue) junctions are observed. Boxes indicate drugs targeting PI3K/mTOR and ERK MAPK signaling.

Supplementary Figure 12 DJE section of DJEC DB showing summary statistics table, gene-wise splice plots and gene model plots of *NUMB* in TCGA BRCA. The two upregulated junctions indicating the inclusion of exon 12 in *NUMB* are shown in red within the gene-wise splice plot and the selected junction in the summary statistics table is also highlighted within the gene model plot (Panel 1 highlights the selection option section. Panel 2 contains the summary statistics table. Panel 3 and 4 show the gene-wise splice plot and the domain-annotated gene model plot, respectively).

Supplementary Figure 13 Downregulation of exon 19 in *VCL* illustrated by DJE section in DJEC DB. Exon inclusion junctions are shown in blue within the gene-wise splice plot and the selected downregulated junction in the summary statistics table is also shown within the gene model plot. CESC TCGA results are shown as example (Panel 1 highlights the selection option section. Panel 2 contains the summary statistics table. Panel 3 and 4 show the gene-wise splice plot and the domain-annotated gene model plot, respectively).

Supplementary Figure 14 Cancer-specific upregulation of exon 12 in *MAP3K7* as shown in DJEC DB. Exon inclusion and exclusion junctions are highlighted in red and blue respectively within the gene-wise splice plot. The selected upregulated junction in the summary statistics is illustrated within the gene model plot. COADREAD TCGA results are shown as example (Panel 1 highlights the selection option section. Panel 2 contains the summary statistics table. Panel 3 and 4 show the gene-wise splice plot and the domain-annotated gene model plot, respectively).

Supplementary Figure 15 Cancer-specific alternative splicing in *EXOC1* as shown in DJEC DB. Junctions indicating the upregulation of exon 11 in *EXOC1* are shown in red within the gene-wise splice plot. The selected upregulated junction in the summary statistics is illustrated within the gene model plot. LUAD TCGA results are shown as example (Panel 1 highlights the selection option section. Panel 2 contains the summary statistics table. Panel 3 and 4 show the gene-wise splice plot and the domain-annotated gene model plot, respectively) (Panel 1 highlights the selection option section. Panel 2 contains the summary statistics table. Panel 3 and 4 show the gene-wise splice plot and the domain-annotated gene model plot, respectively).

Supplementary Figure 16 Significant associations using *Matrix eQTL* methods between *CTNND1* exon 20 inclusion event and genomic alterations in TCGA are shown within the JT section of DJEC DB. Selecting “Associations with Genomic Alterations” and “BRCA” tumor type within the selection panel (Panel 1), followed by “CTNND1” gene ID browsing within the summary statistics table (Panel 2) displays the significant association to *TP53* mutation. Box plots show decreased exon junction expression in the presence of TP53 mutation (MUT), compared to wild-type (WT) tumor samples (Panel 3). amplification of *CCND1* gene and epigenetic silencing of *CDKN2A* are also significantly associated to *CTNND1* alternative splicing event in TCGA STES (Panel 4).
