# Supplementary figures and images for: DJExpress: An Integrated Application for Differential Splicing Analysis and Visualization
Source: Front Bioinform. 2022 Feb 24;2:786898. doi: 10.3389/fbinf.2022.786898 (PMC9580925; doi:10.3389/fbinf.2022.786898)

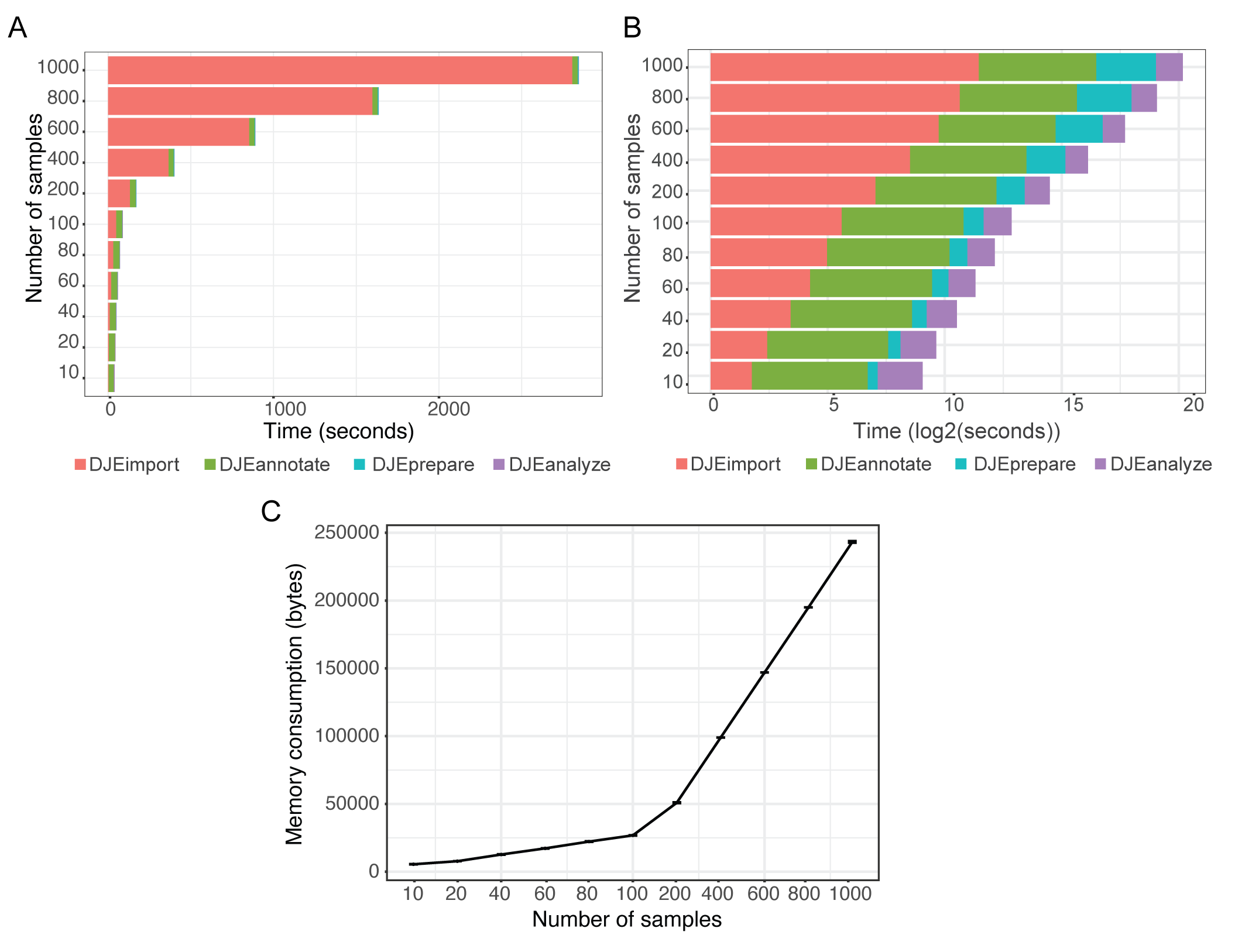

Supplement: Supplementary file 1 [file DataSheet1.zip › Supplementary Figure 1.tif]

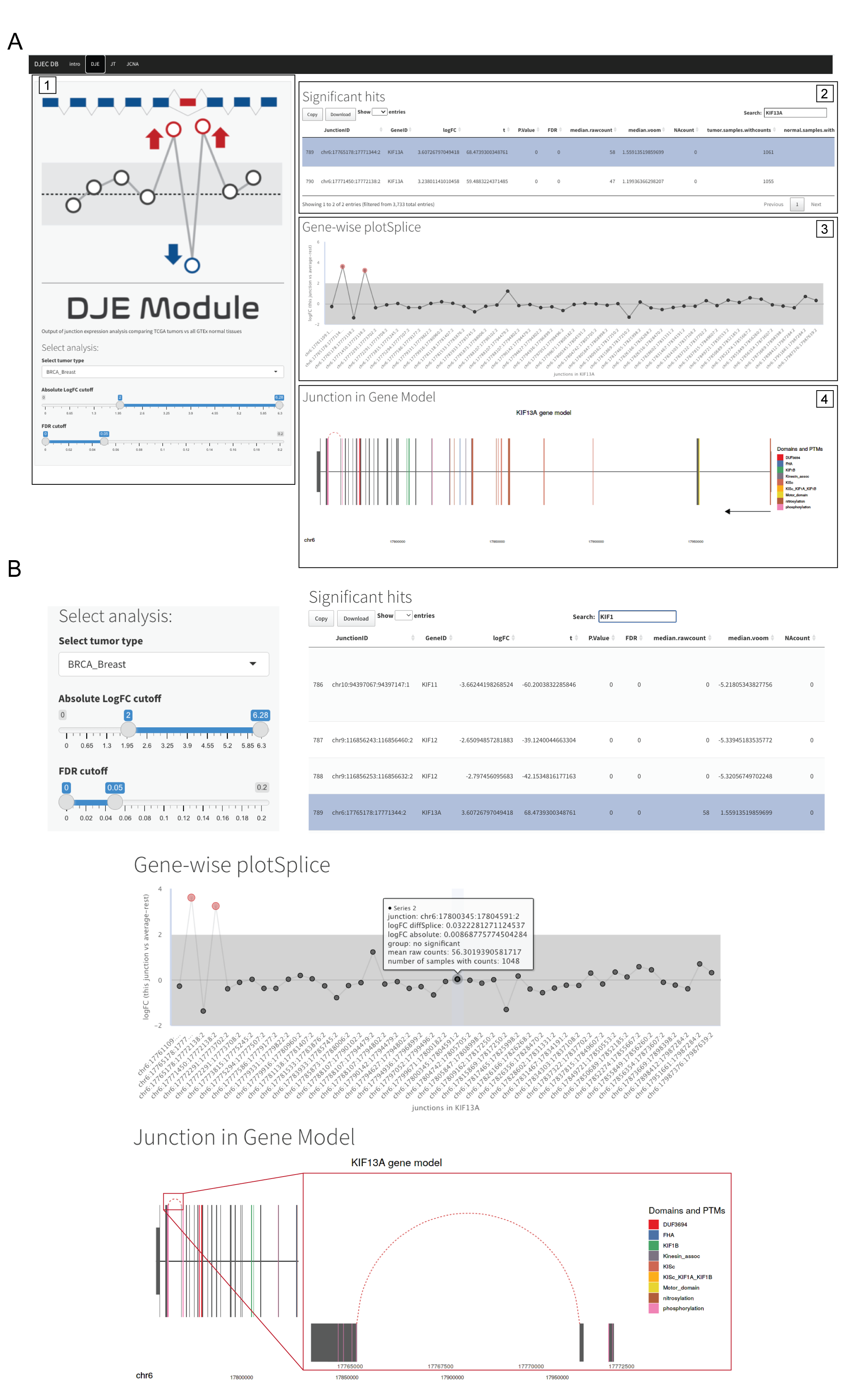

Supplement: Supplementary file 1 [file DataSheet1.zip › Supplementary Figure 2.tif]

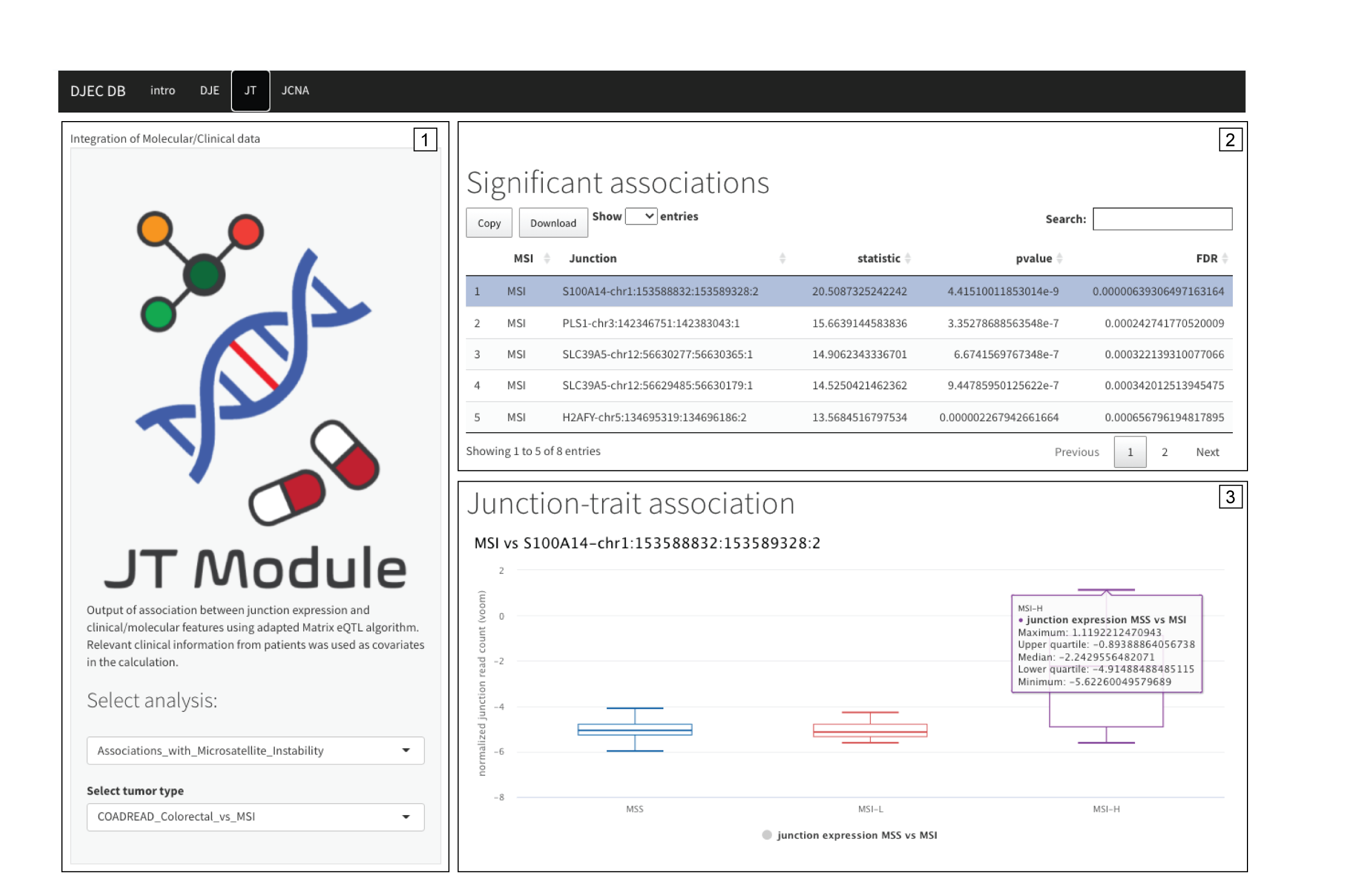

Supplement: Supplementary file 1 [file DataSheet1.zip › Supplementary Figure 3.tif]

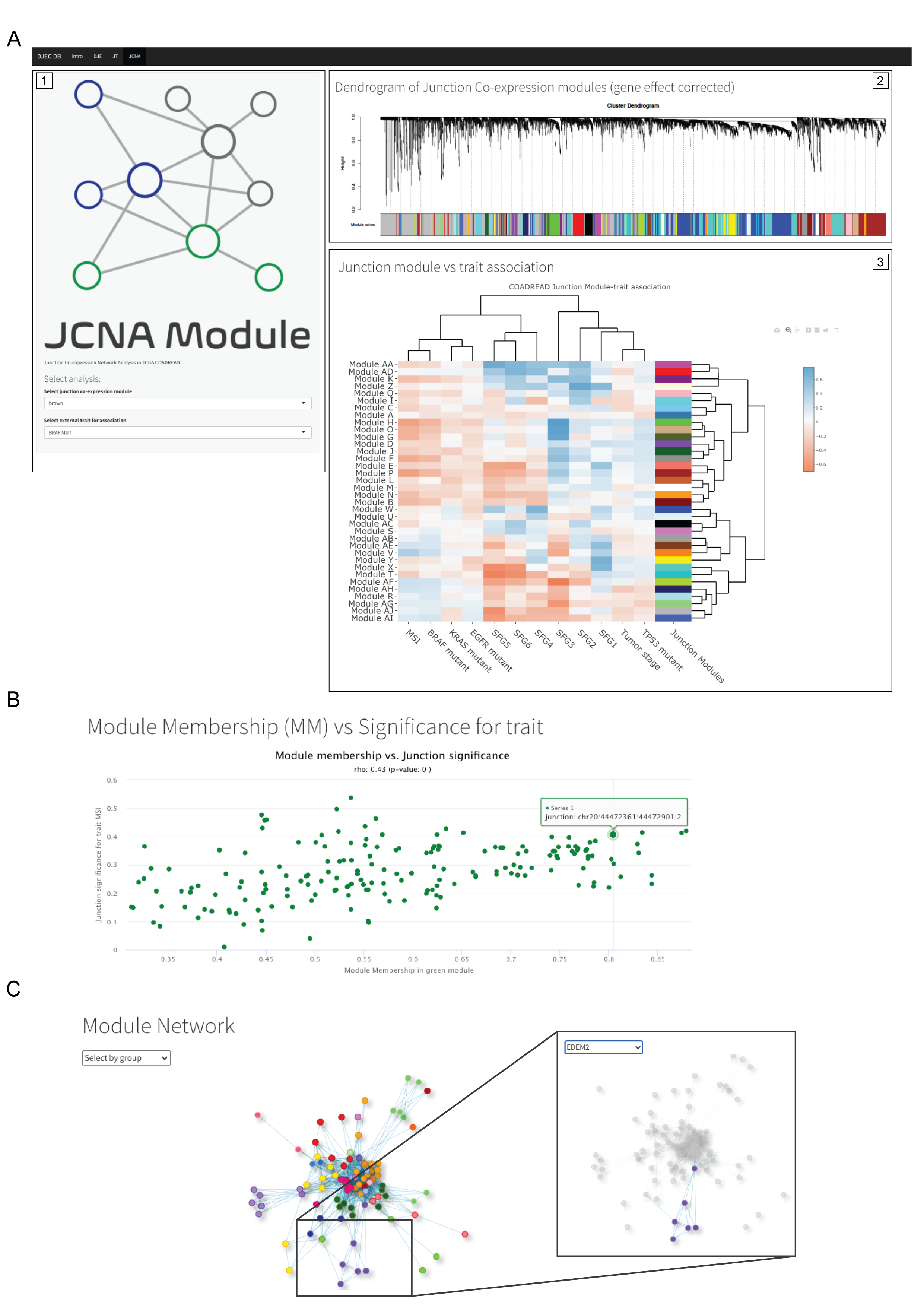

Supplement: Supplementary file 1 [file DataSheet1.zip › Supplementary Figure 4.tif]

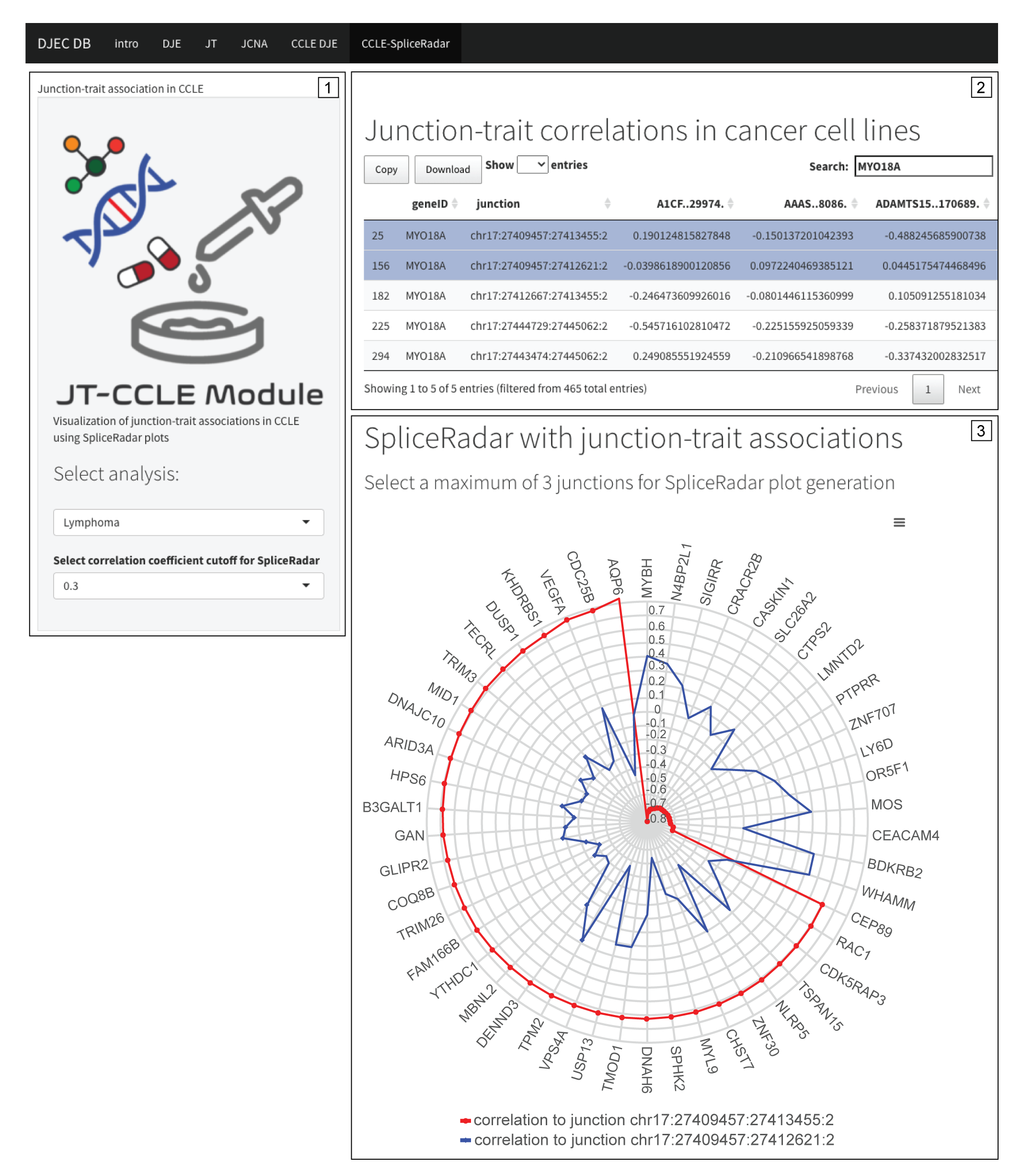

Supplement: Supplementary file 1 [file DataSheet1.zip › Supplementary Figure 5.tif]

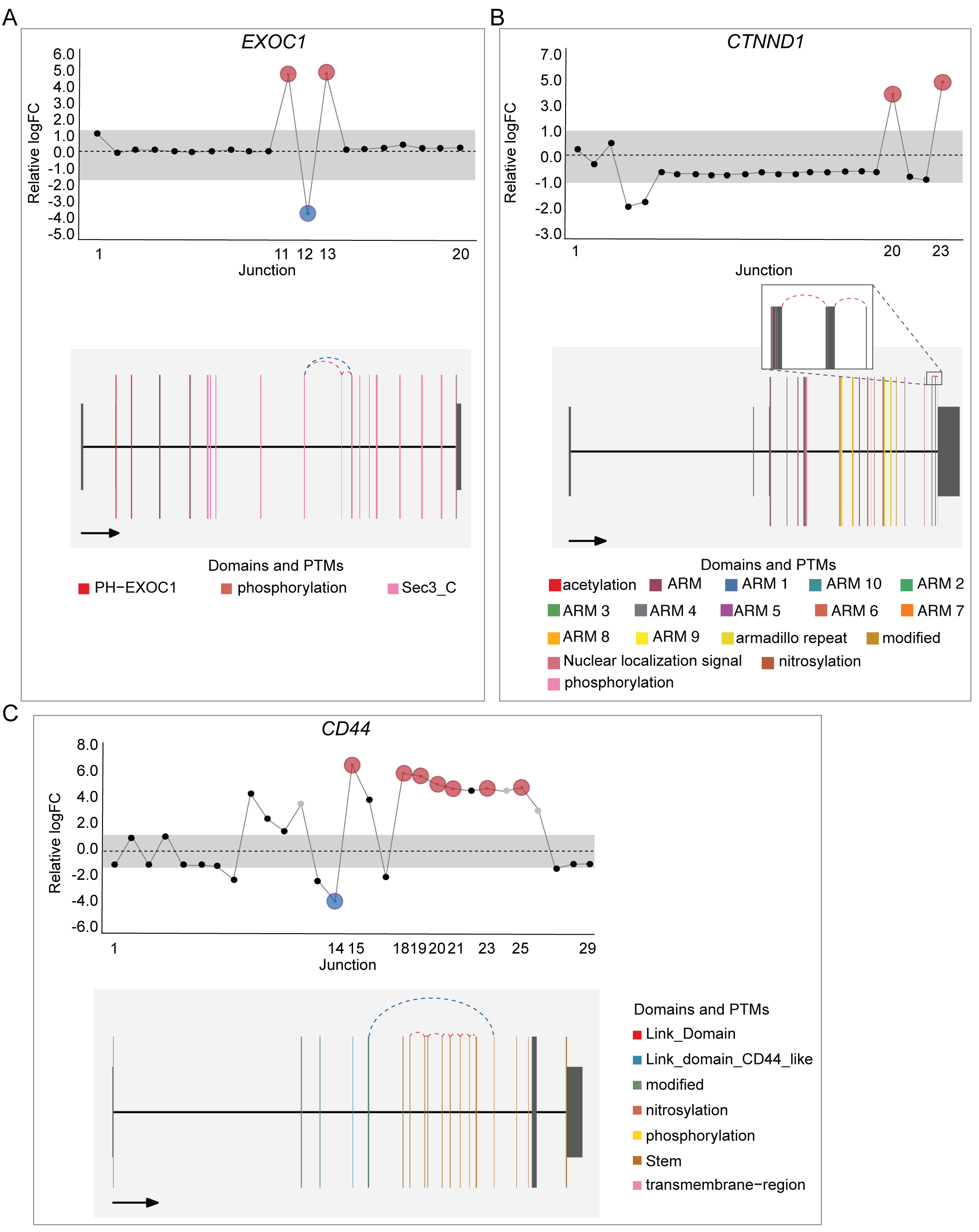

Supplement: Supplementary file 1 [file DataSheet1.zip › Supplementary Figure 6.tif]

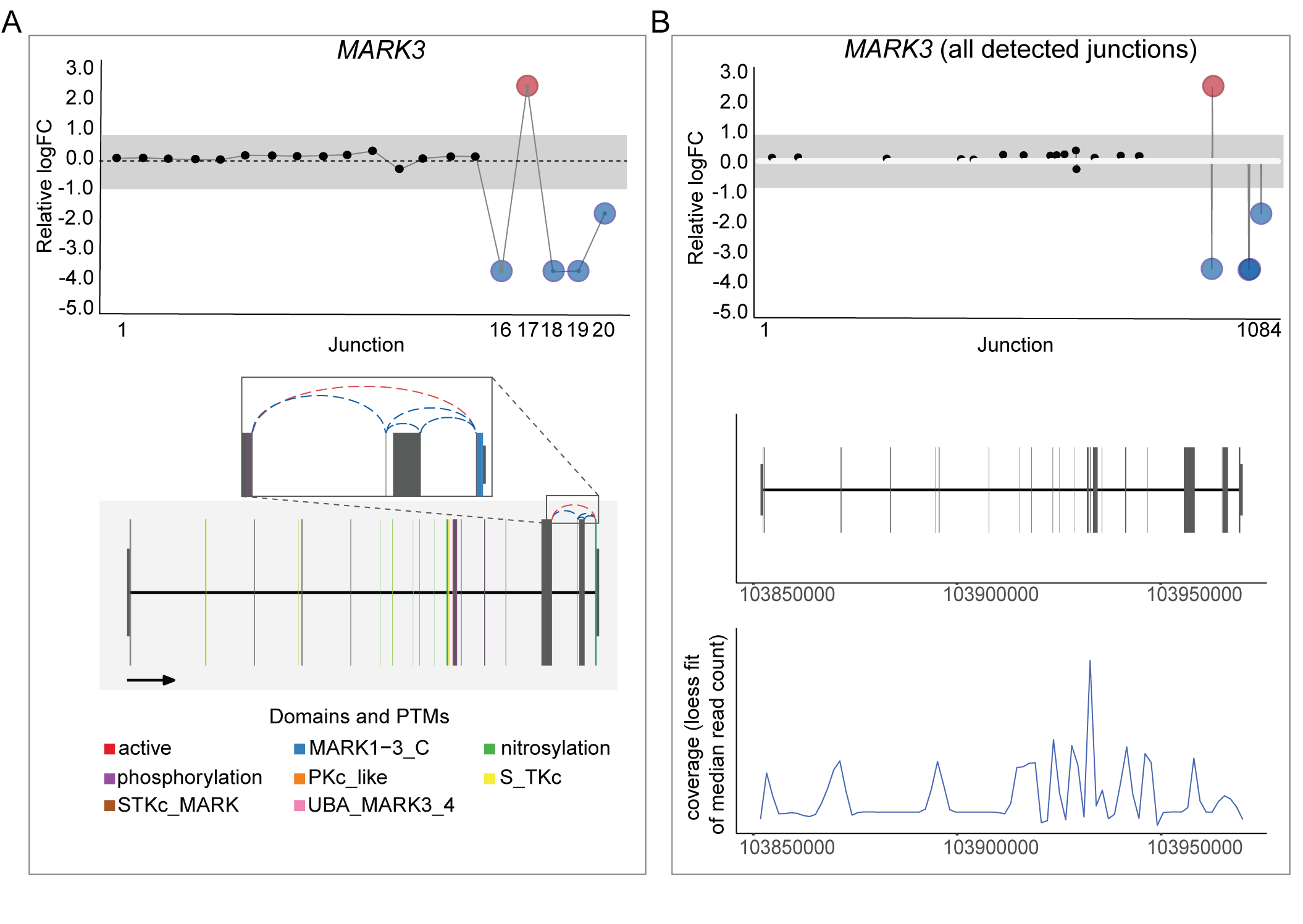

Supplement: Supplementary file 1 [file DataSheet1.zip › Supplementary Figure 7.tif]

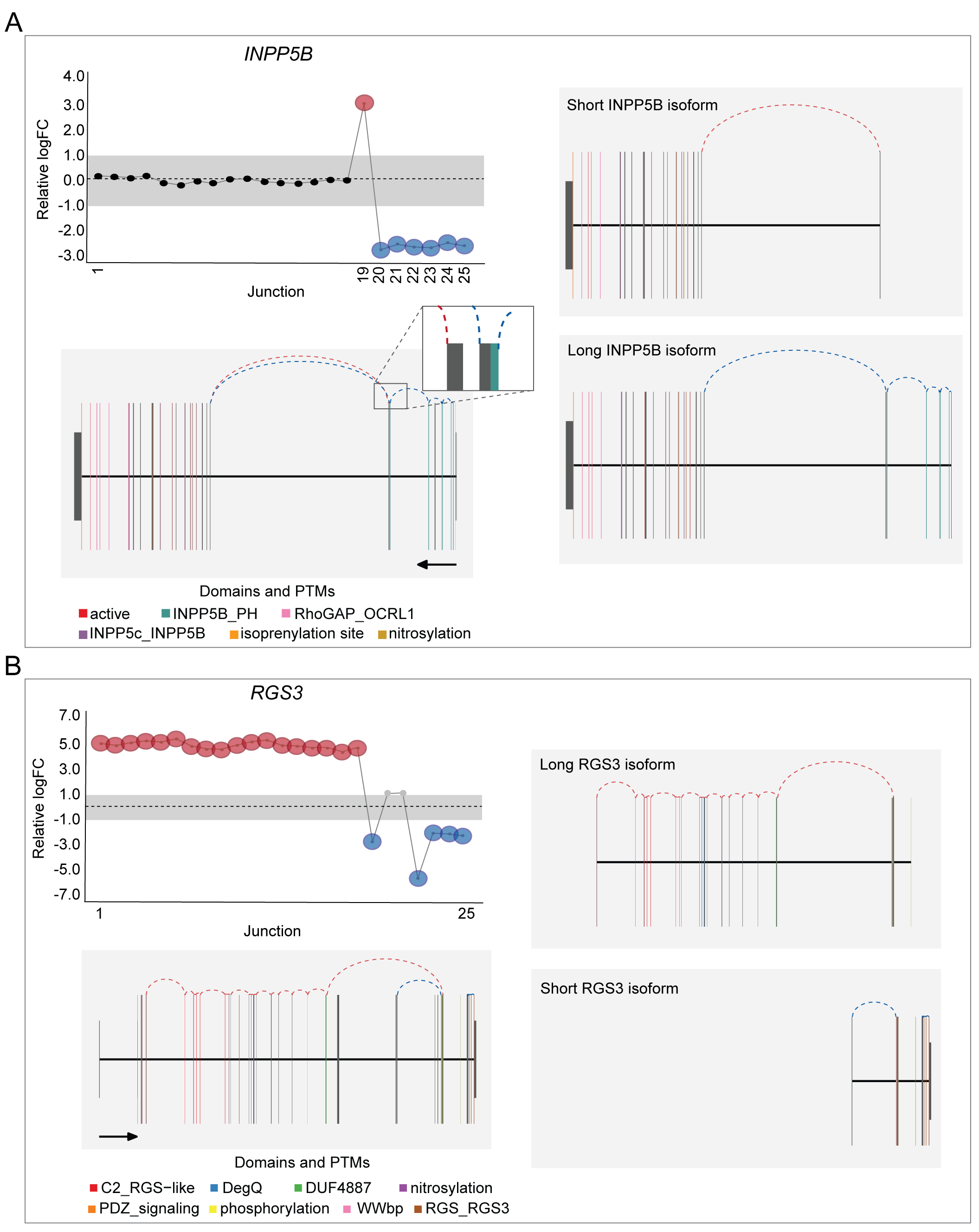

Supplement: Supplementary file 1 [file DataSheet1.zip › Supplementary Figure 8.tif]

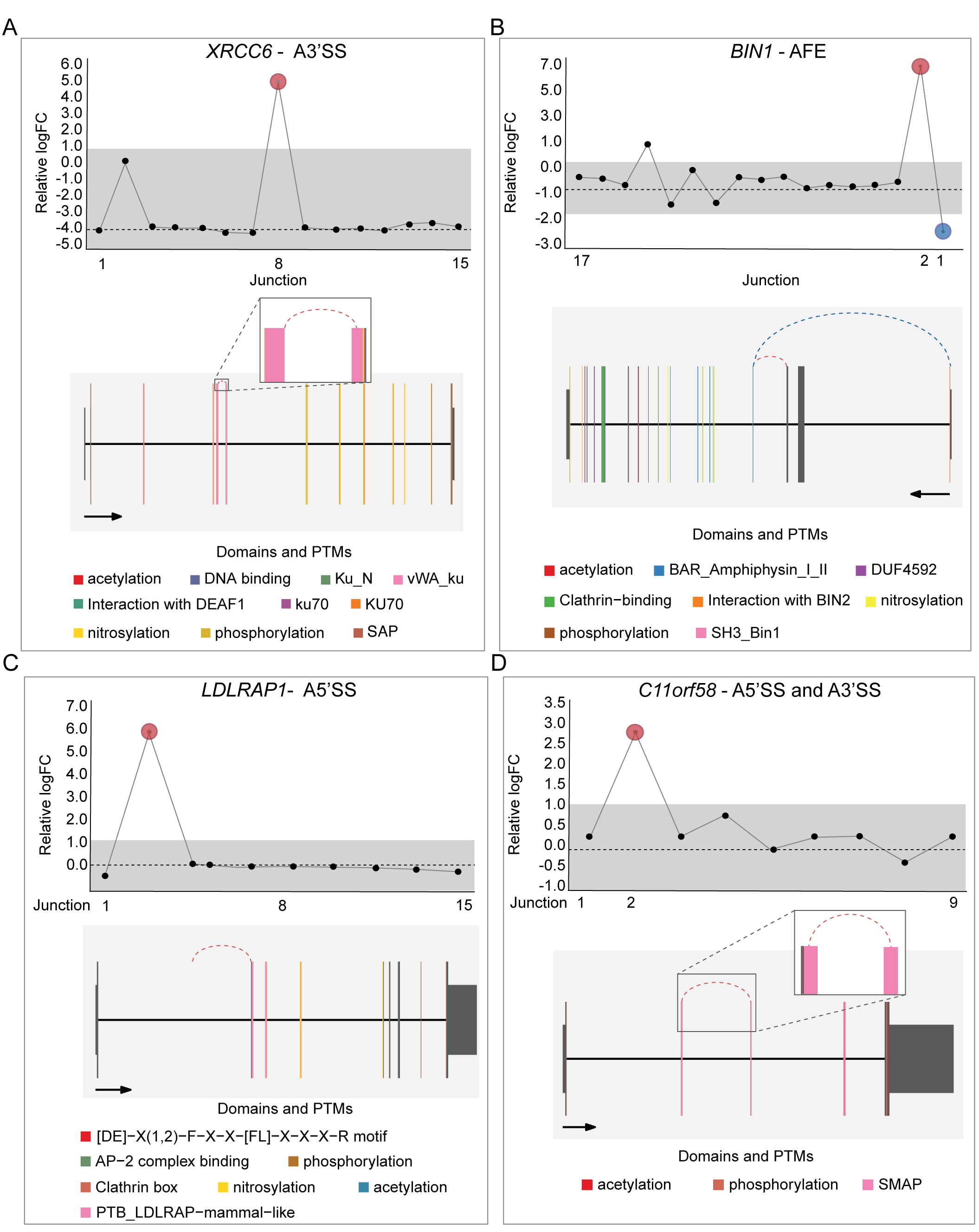

Supplement: Supplementary file 1 [file DataSheet1.zip › Supplementary Figure 9.tif]

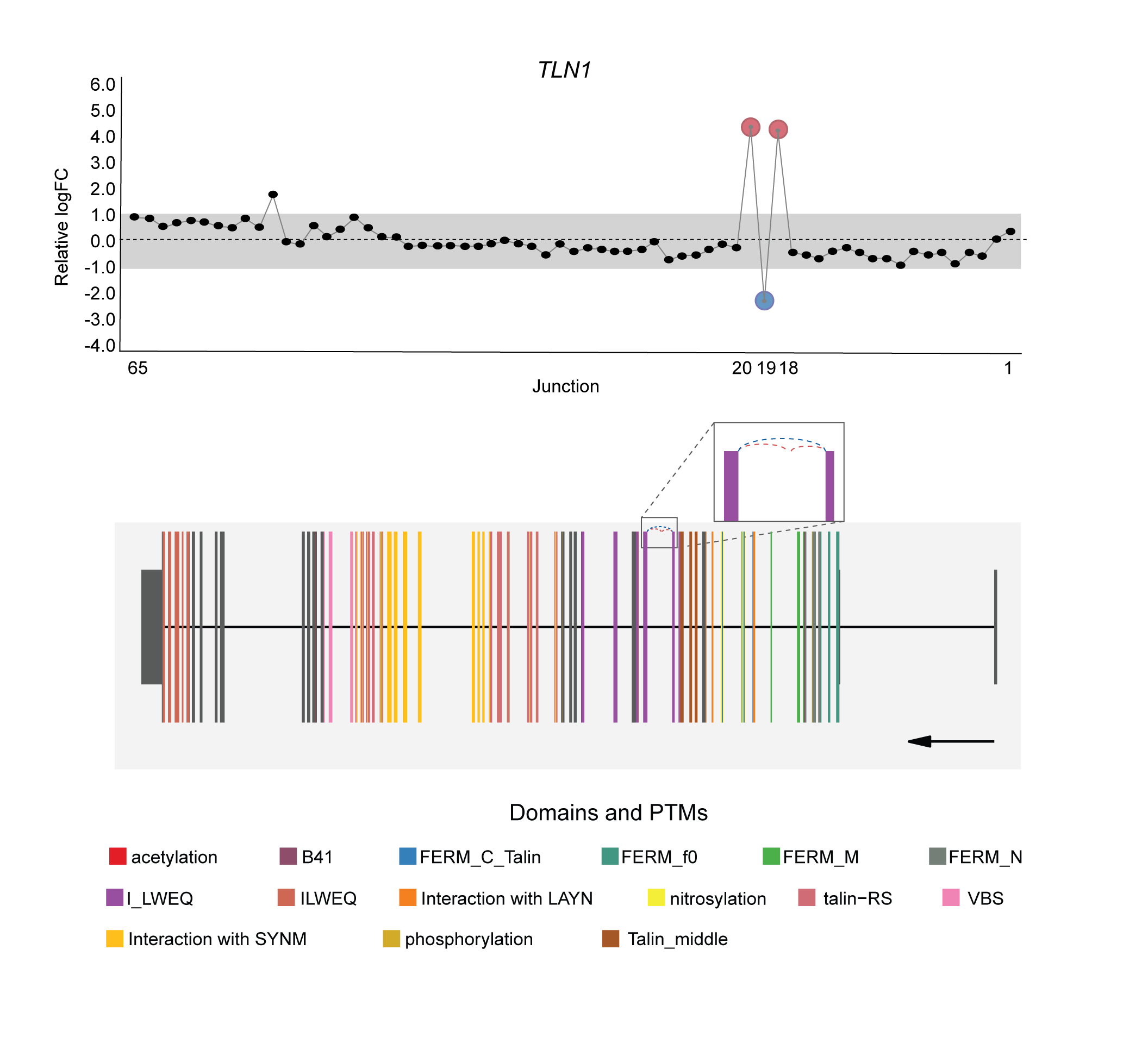

Supplement: Supplementary file 1 [file DataSheet1.zip › Supplementary Figure 10.tif]

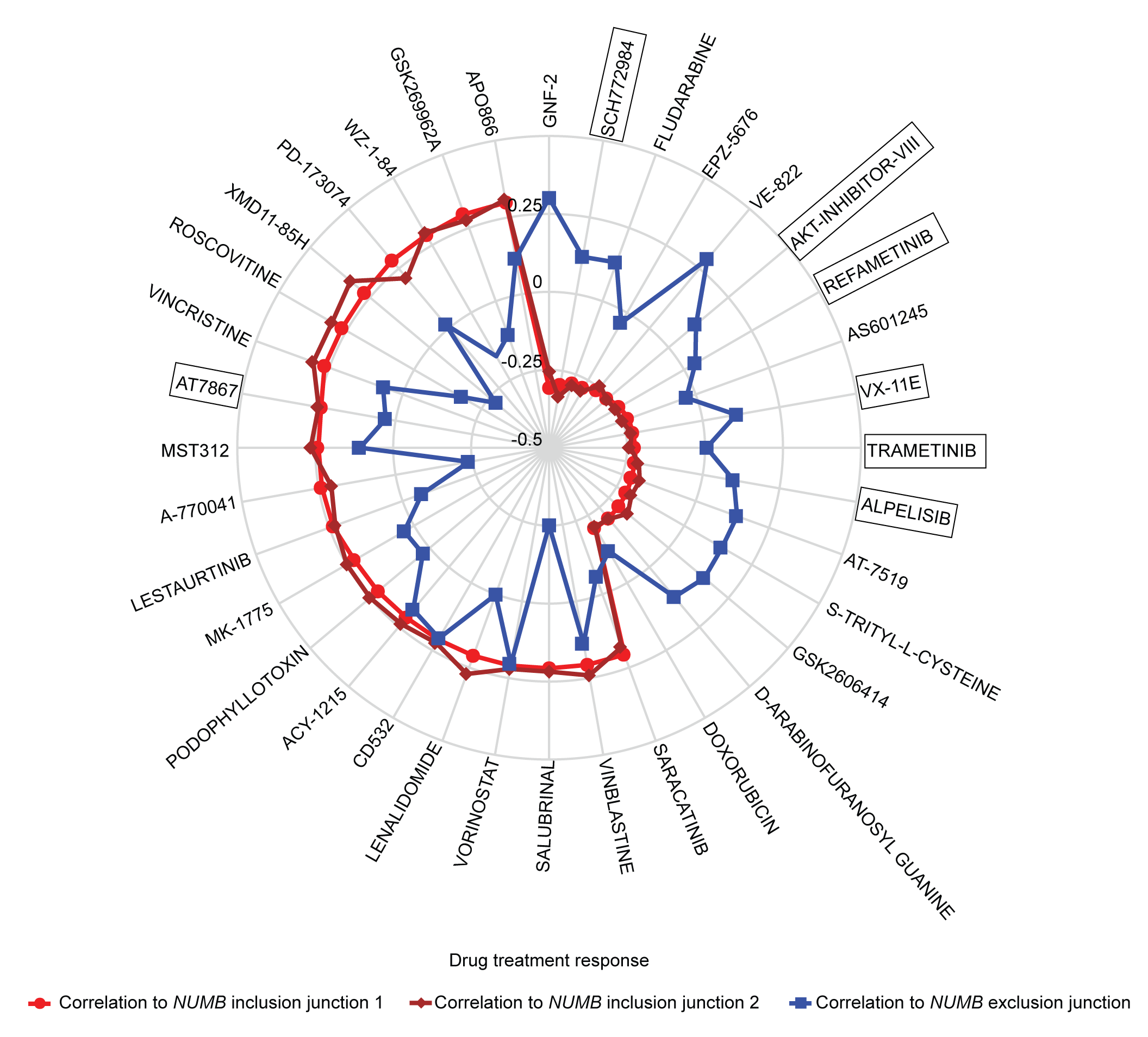

Supplement: Supplementary file 1 [file DataSheet1.zip › Supplementary Figure 11.tif]

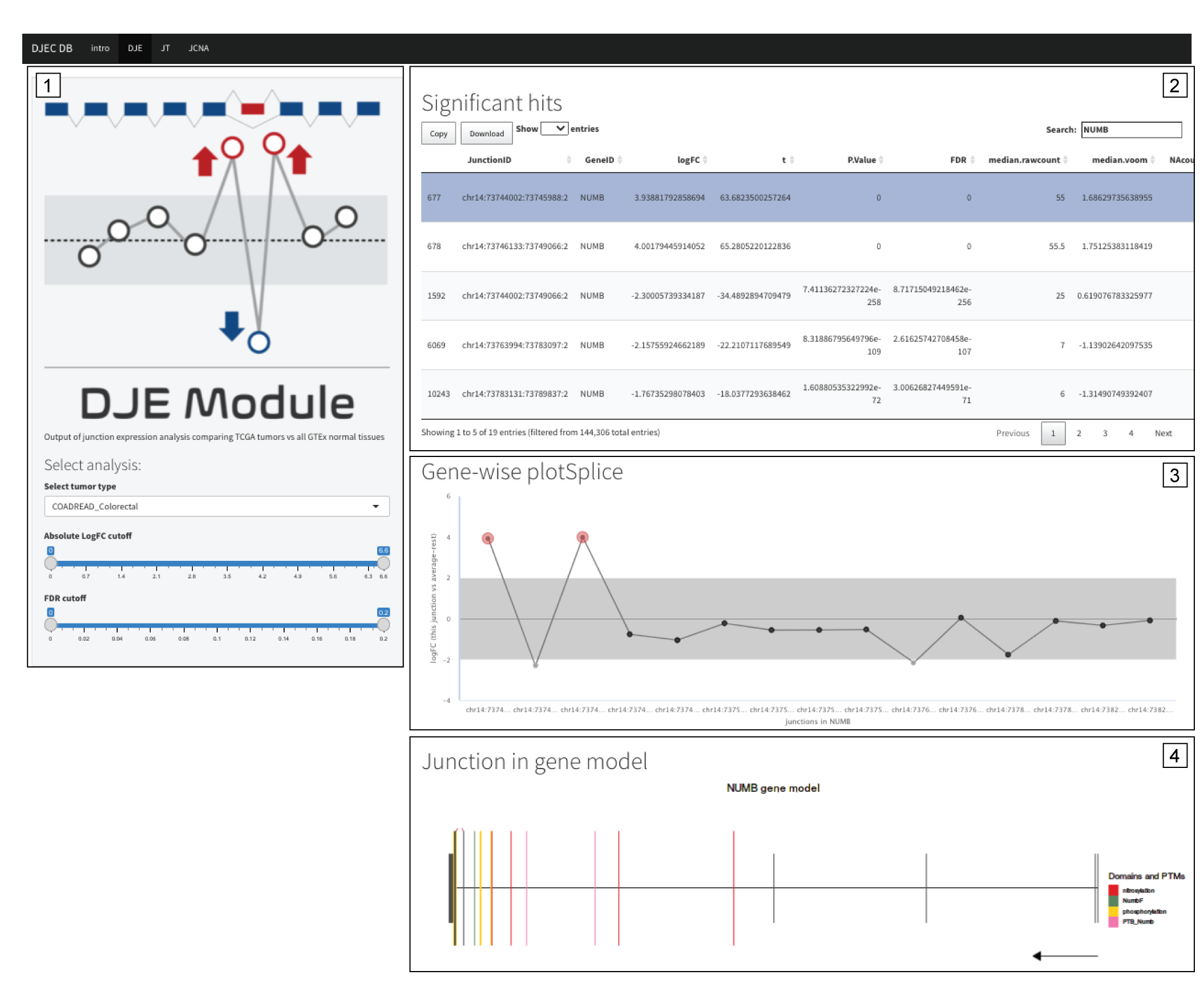

Supplement: Supplementary file 1 [file DataSheet1.zip › Supplementary Figure 12.tif]

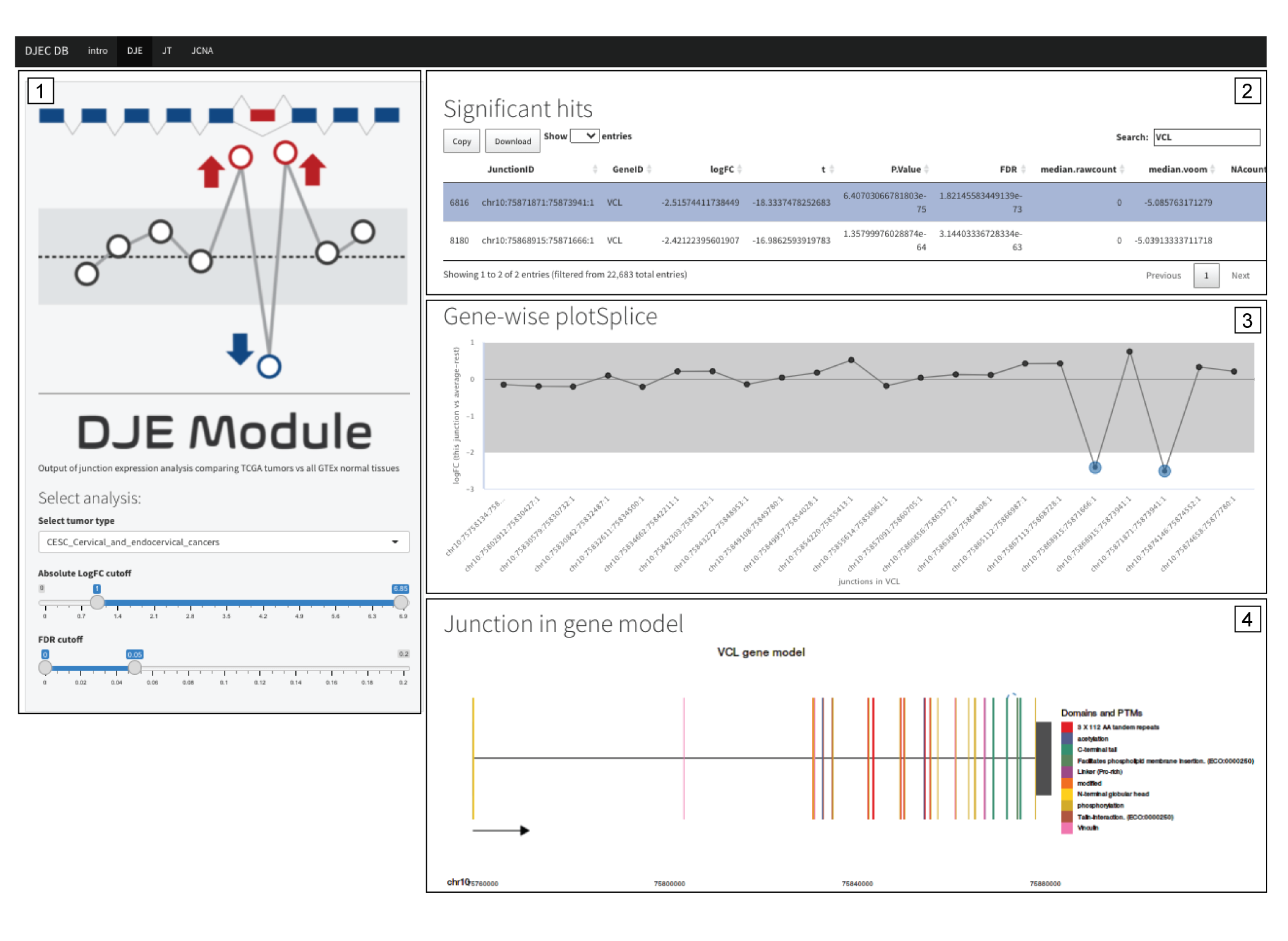

Supplement: Supplementary file 1 [file DataSheet1.zip › Supplementary Figure 13.tif]

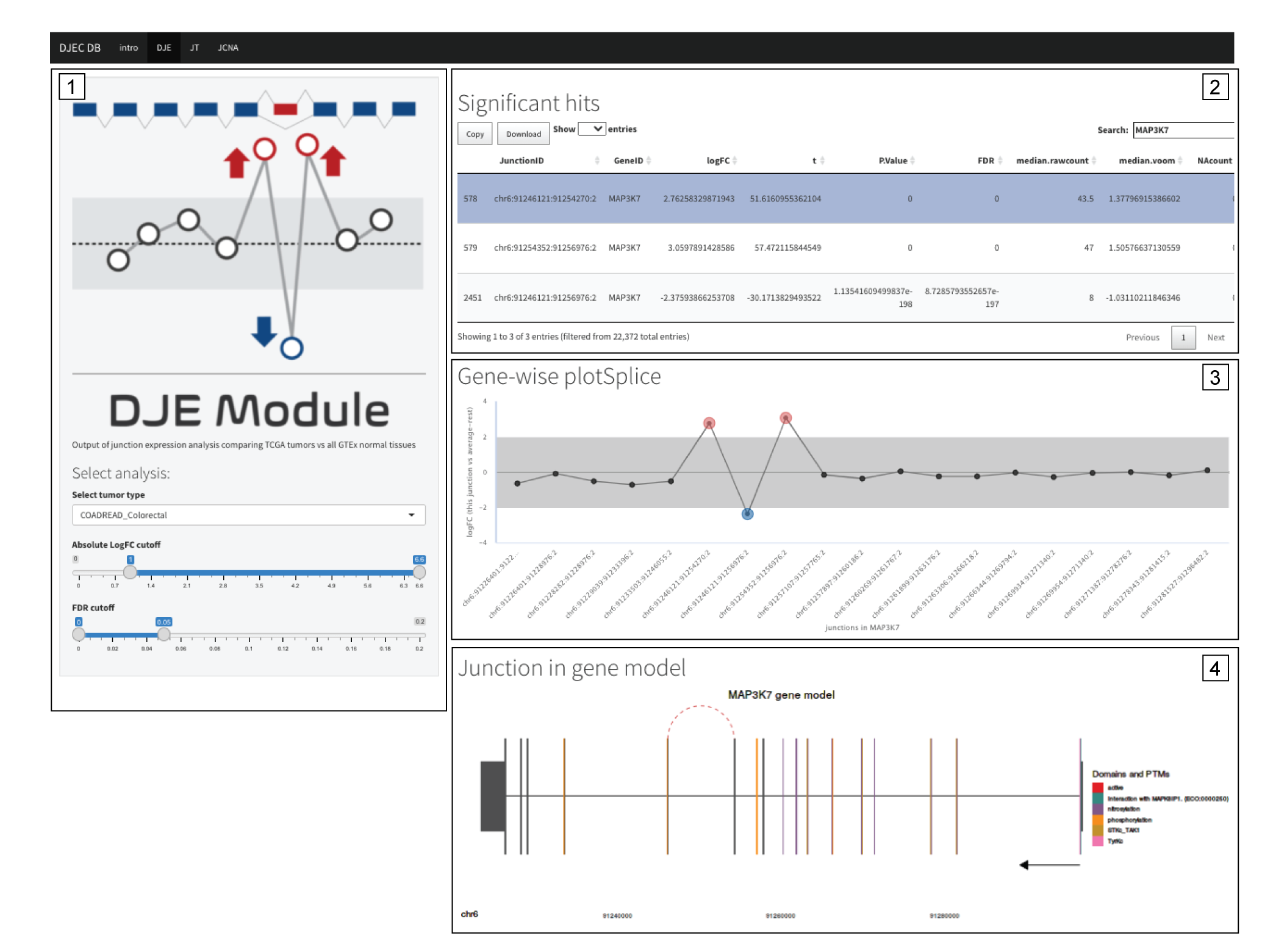

Supplement: Supplementary file 1 [file DataSheet1.zip › Supplementary Figure 14.tif]

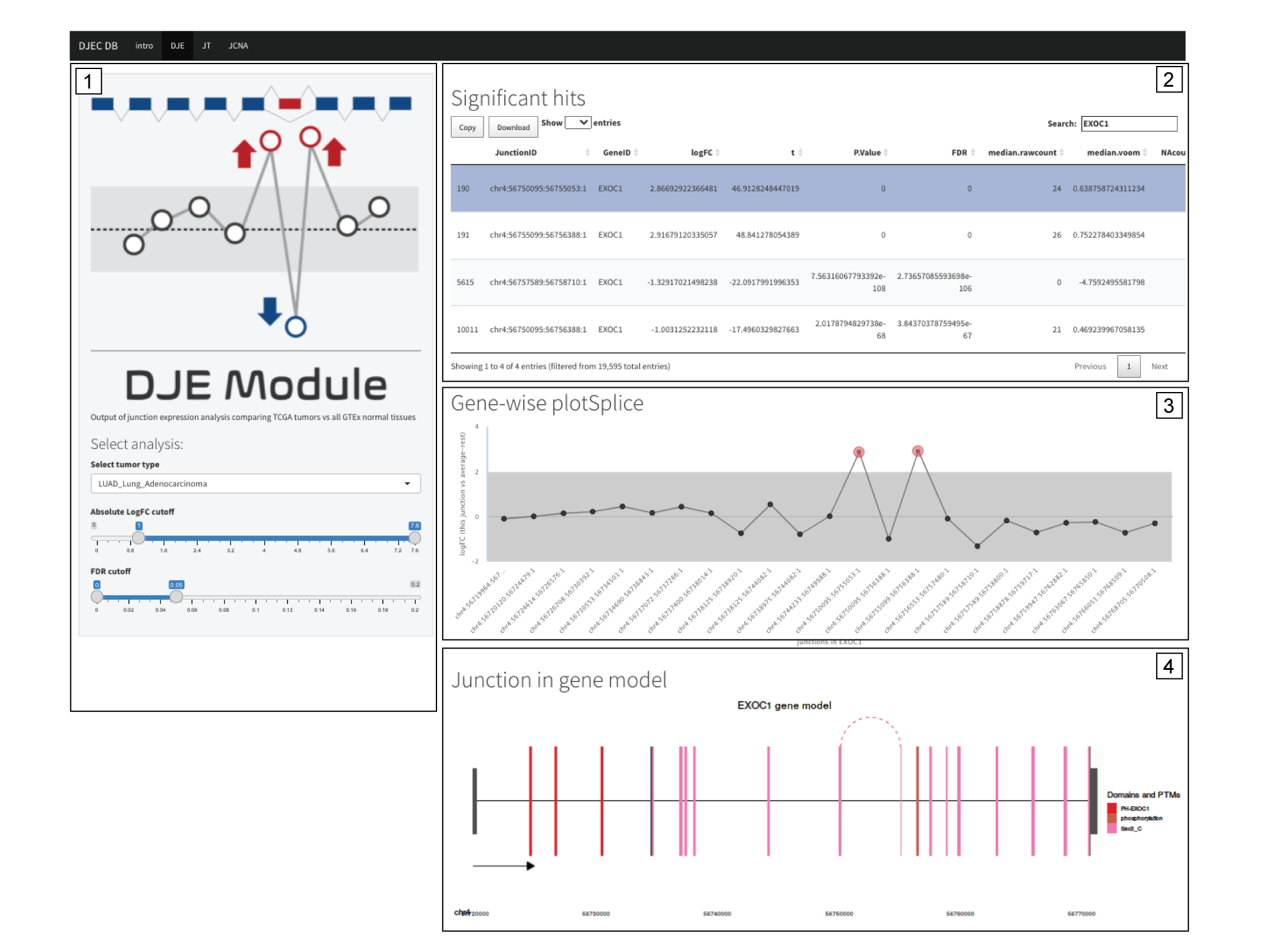

Supplement: Supplementary file 1 [file DataSheet1.zip › Supplementary Figure 15.tif]

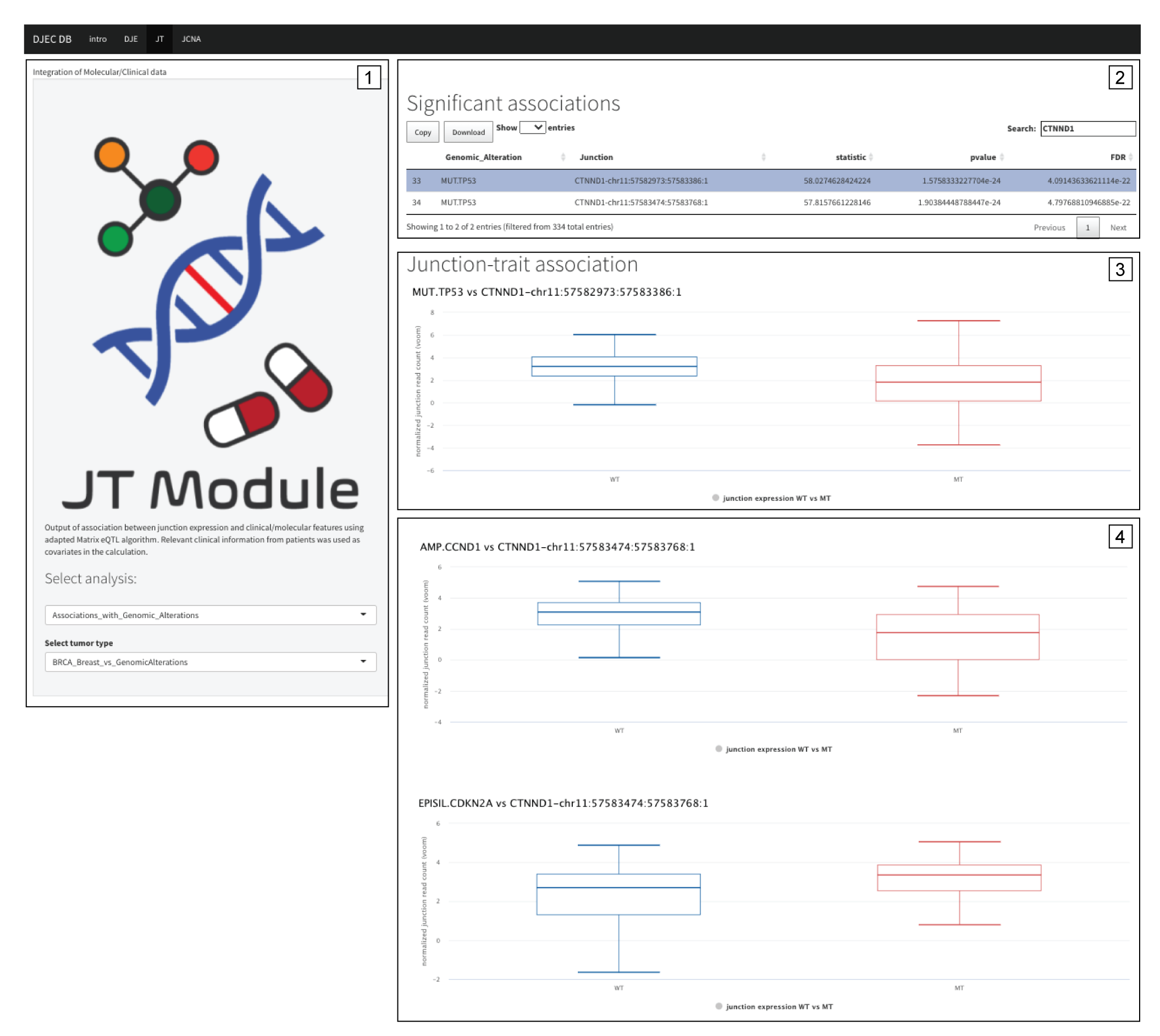

Supplement: Supplementary file 1 [file DataSheet1.zip › Supplementary Figure 16.tif]
